# Machine learning discriminates P2X7-mediated intracellular calcium sparks in human-induced pluripotent stem cell-derived neural stem cells

**DOI:** 10.1038/s41598-023-39846-4

**Published:** 2023-08-04

**Authors:** Yuki Hanafusa, Akira Shiraishi, Fumiyuki Hattori

**Affiliations:** 1 Innovative Regenerative Medicine, Graduate School of Medicine, Kansai Medical University, Osaka, Japan; 2 Group Quality Assurance Division, Safety Science Institute, Suntory Holdings Ltd., Tokyo, Japan; 3 Division of Integrative Biomolecular Function, Bioorganic Research Institute, Suntory Foundation for Life Sciences, Kyoto, Japan

**Keywords:** Cell biology, Computational biology and bioinformatics, Neuroscience, Stem cells, Health care

## Abstract

Adenosine triphosphate (ATP) is an extracellular signaling molecule that mainly affects the pathophysiological situation in the body and can be sensed by purinergic receptors, including ionotropic P2X7. Neuronal stem cells (NSCs) remain in adult neuronal tissues and can contribute to physiological processes via activation by evoked pathophysiological situations. In this study, we revealed that human-induced pluripotent stem cell-derived NSCs (iNSCs) have ATP-sensing ability primarily via the purinergic and ionotropic receptor P2X7. Next, to develop a machine learning (ML)-based screening system for food-derived neuronal effective substances and their effective doses, we collected ATP-triggered calcium responses of iNSCs pretreated with several substances and doses. Finally, we discovered that ML was performed using composite images, each containing nine waveform images, to achieve a better ML model (MLM) with higher precision. Our MLM can correctly sort subtle unidentified changes in waveforms produced by pretreated iNSCs with each substance and/or dose into the positive group, with common mRNA expression changes belonging to the gene ontology signatures.

## Introduction

The brain is known to be the most complex and flexible organ in the body. The development of the neural network within the fetal brain is prone to be affected by chemicals that pass through the placental barrier^1,2^. After birth, the human brain continues to grow and develop functional neural networks that receive external stimulation as meaningful information. However, postnatal exposure to neurotoxic substances such as polychlorinated biphenyl can have negative effects on mental and motor development^3^. Even in the adult brain, established essential neural networks need to be maintained, and new networks need to be developed until death^4^. Recent studies have revealed that the human adult brain contains neural stem cells (NSCs) for de novo neurogenesis for regeneration and/or for establishing new networks^5^. To maintain a balance between homeostasis and changes in neuronal networks, it is necessary to study the effective neuronal doses of substances taken into the body.

Food and drinks are the most common sources of nutrients and neuronal substances, including neurotoxins^6^. In modern industrialized countries, several new foods and supplements with poor intake experiences are entering the market. For example, sibutramine-containing slimming supplements can cause severe health problems^7^. In addition to pure neurotoxins, it is important to know the effective doses of daily food containing substances that affect neurons such as caffeine, alcohol, and theanine.

Neuronal effects have mainly been tested in animals. However, to address concerns about animal welfare, alternative methods for predicting neurotoxicity must be developed. Human-induced pluripotent stem cells (hiPSCs) are an ideal resource for toxicological screening systems because they can provide various types of neuronal cells with fewer ethical issues. Many reports have shown that hiPSC-derived neurons are useful for evaluating and characterizing the neurotoxicity of specific substances related to cell death or synaptogenesis^8,9^. However, to date, no screening system can dose-dependently segregate general food-containing substances into groups with or without neuronal effects.

Adenosine triphosphate (ATP) is the main purinergic messenger that serves as an indication of brain damage^10^. The concentration of ATP reflects the severity of the damage, as the damaged cells release ATP uncontrollably. Extracellular ATP can lead to a “damage-induced damage” cycle through the induction of direct neuronal cell death and microglial inflammatory responses^11^. ATP is sensed by purinergic receptors, of which P2X7 is widely expressed in excitable cells, including human pluripotent stem cell-derived neuronal progenitor cells, astrocytes, microglia-like cells, and neurons^12^. However, the functionality of P2X7 in human iNSCs remains unclear.

P2X7 is an ionotropic receptor. When activated by ATP, it opens a larger cationic channel permeating potassium, sodium, and calcium ions rather than other subtypes^13^. Therefore, P2X7 is believed to play an important role in pathological conditions, such as cell death, inflammation, and excitatory neural injuries. Ionotropic P2X7 activation in neurons causes calcium sparks with characteristics of high frequency, tapered high magnitude, and short duration, which are distinguished from calcium waves characteristically and mechanistically^14^. Calcium sparks may be caused by P2X7-driven membrane potential changes that directly activate voltage-gated L-type calcium channels, followed by the activation of calcium-induced calcium release from the endoplasmic reticulum via activation of the ryanodine receptor channel.

Machine learning has been widely used in the field of medical diagnostics. However, its application to toxicological screening systems as a replacement for in vitro and in vivo tests remains challenging. Kowalczewski et al.^15^ reported that a machine-learning model can distinguish the responses of hiPSC-derived cardiomyocytes to representative arrhythmogenic agents, isoproterenol, verapamil, and cisapride, based on known calcium wave parameters. Monzel et al.^16^ developed a machine-learning model that can discriminate between dopaminergic neuron-specific toxin 6-hydroxydopamine-treated hiPSC-derived neuronal organoids.

In this study, we confirmed that functional P2X7 is expressed in human-induced pluripotent stem cell-derived NSCs (iNSCs) and investigated the effect of pretreatment with known neurotoxic and dose-dependent neuronal effective compounds by global mRNA expression profiling. Next, we developed a machine learning (ML) model using raw calcium spark images containing undefined potential effects by chemical treatment using a new methodology. The developed ML model could accurately segregate intratest-group data into neuronal effecting or non/less neuronal effecting groups and successfully predict noneducated neuronal effecting compounds as members of the neuronal effecting group.

## Results

### Study design

**Figure 1:**
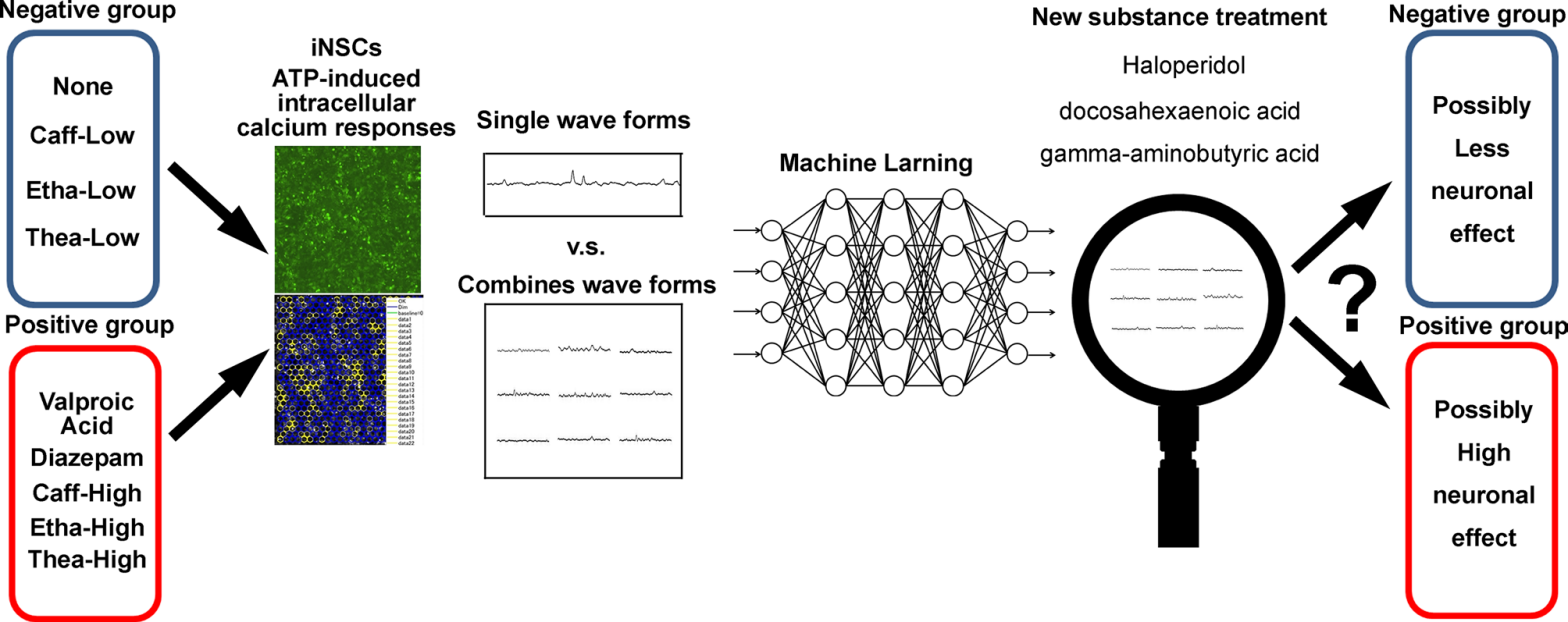
Schematic drawing of the whole study design and aim.

We designed the entire study, as shown in Fig. 1. From the literature, we determined highly effective concentrations as high doses and less effective concentrations as low doses to neuronal cells^17–20^. The concentrations used for treatment are listed in Table 1.

**Table 1:** Substance names and doses in the groups.

		Concentration (μM)
Negative	None	–
	Caffeine-low	6
	Ethanol-low	2400
	Theanine-low	10
Positive	Caffeine-high	150
	Ethanol-high	60,000
	Theanine-high	250
	Valproic acid	1200
	Diazepam	4.4
	Haloperidol	0.053
	DHA	30
	GABA	500

### Confirmation of the differentiation of neural stem cells (iNSCs) and functional expression of P2X7

**Figure 2:**
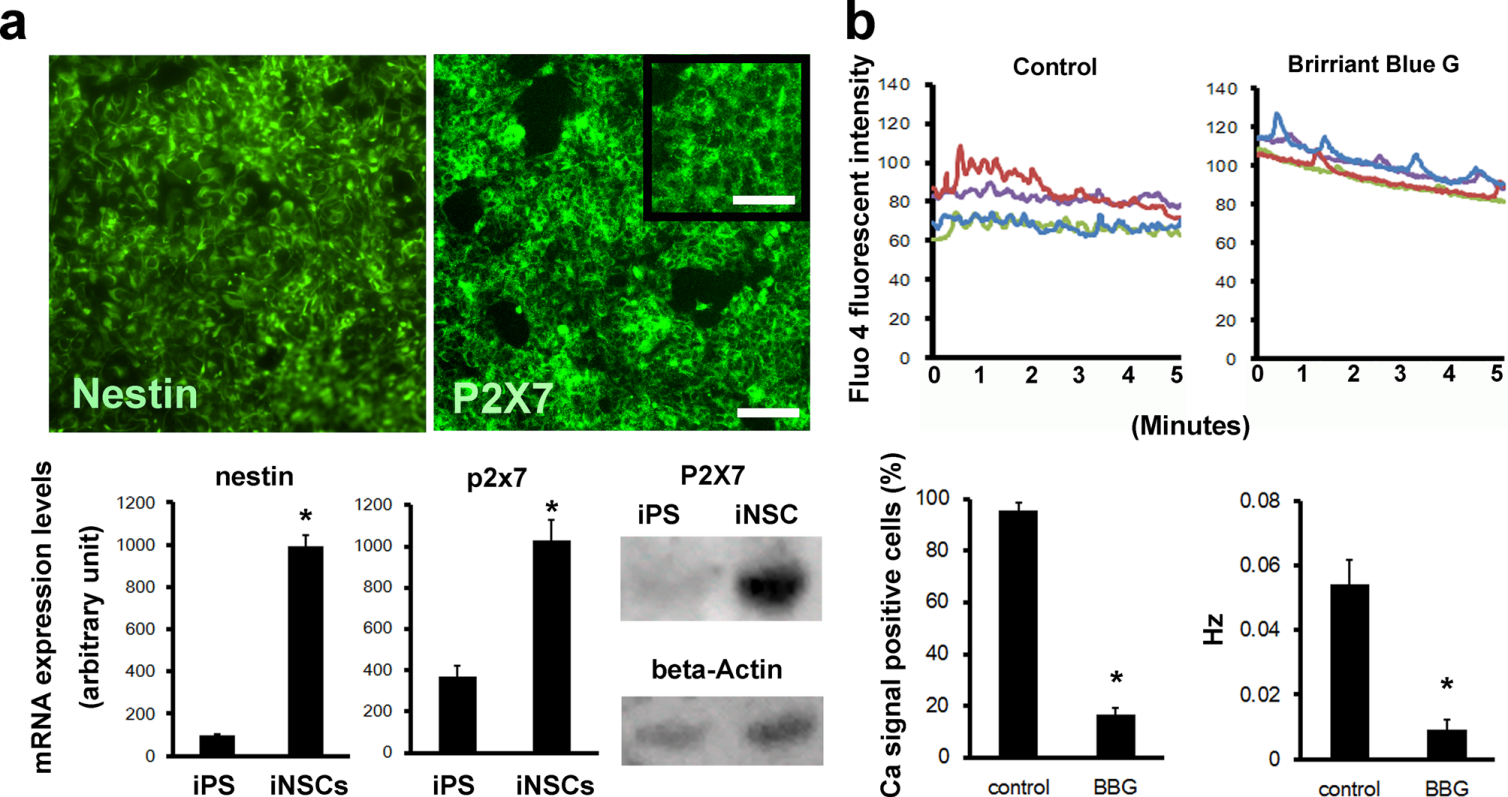
Confirmation of the expression of functional P2X7 in iNSCs. (**a**) Confirmation of the expression of Nestin and P2X7 at the mRNA and protein levels. Scale bars, 100 μm; and 30 μm inset. (**b**) Inhibitory effect of the P2X7-specific antagonist Brilliant Blue G on ATP-induced calcium sparks. Representative calcium sparks of the four cells are shown. The cell fraction of calcium-response-positive cells and their signal frequencies are shown in bar graphs.

We confirmed the successful differentiation of hiPSCs into iNSCs by detecting the expression of Nestin, a neuronal stem cell marker protein, at the mRNA and protein levels (Fig. 2a). Next, we confirmed P2X7 expression at the mRNA and protein levels by quantitative PCR, western blotting, and immunofluorescence staining (Fig. 2a, Supplementary Fig. 1). We also confirmed that iNSCs express other purinergic receptor mRNAs in both the P2X and P2Y families (Supplementary Fig. 2). The calcium spark induced by ATP was completely inhibited by the addition of 20 μM Brilliant Blue G (BBG), a P2X7-specific antagonist^21–24^ (Fig. 2b, Supplementary Movies 1 and 2). We observed an approximately 83% reduction in the number of calcium-responding cells and an approximately 83% reduction in frequency by BBG (Fig. 2B). Please note that because of the blockade of the ionotropic signal from P2X7 by BBG, only calcium waves were detected. We further confirmed the significance of P2X7 in ATP-induced calcium sparks using another P2X7-specific antagonist, JNJ-47965567^12,25,26^, and inhibitor, AZD9056^27^ (Supplementary Fig. 3).

### The effect of the treatments on iNSC global mRNA expression profiles

**Figure 3:**
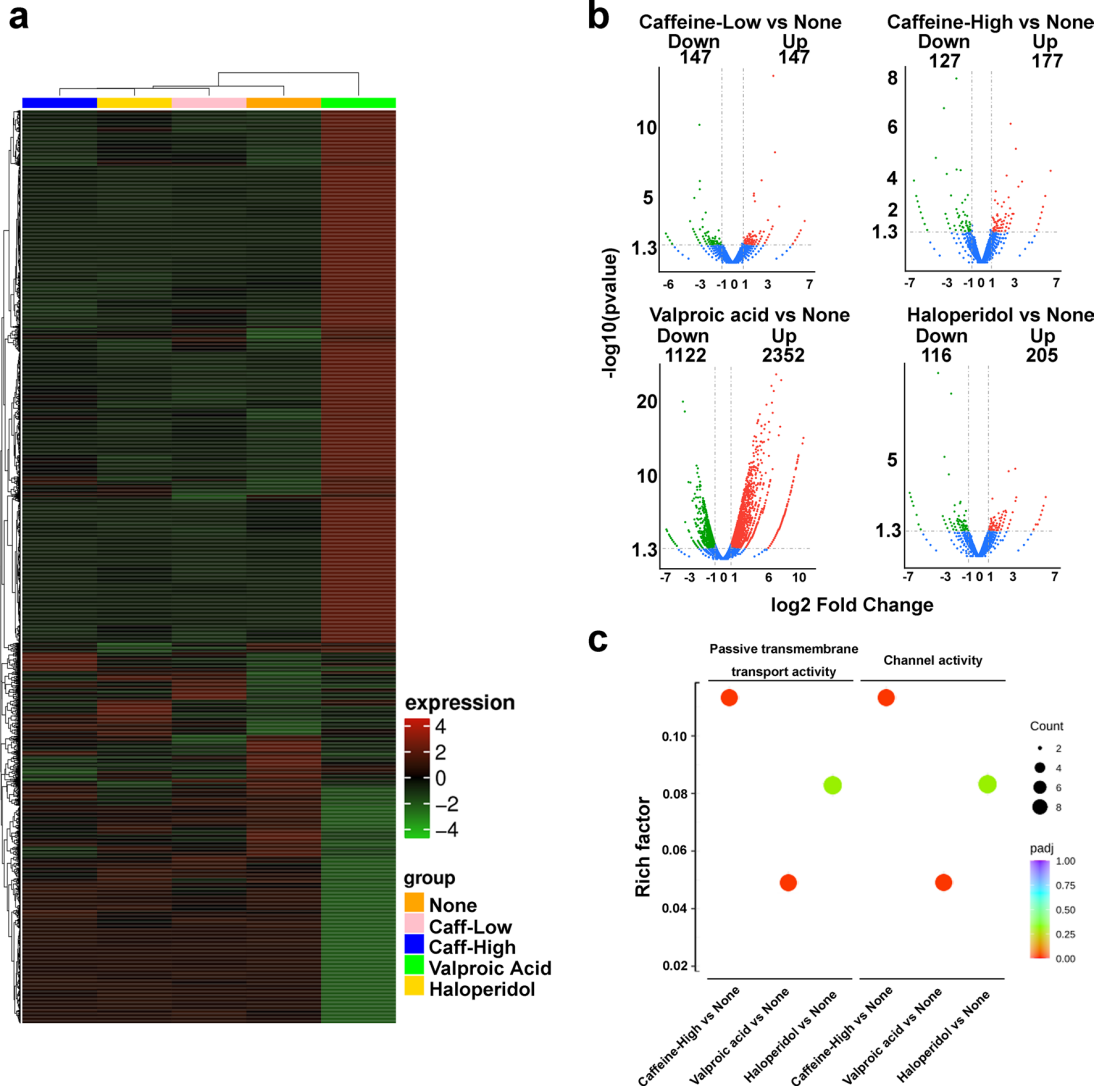
Gene expression changes and common signatures in the positive group. (**a**) Heatmap of the gene expression in iNSCs treated with none (orange), caffeine-low (pink), caffeine high (blue), valproic acid (green), and haloperidol (yellow). (**b**) Volcano plots of differentially expressed genes in RNA-seq compared with none versus caffeine-low, caffeine-high, valproic acid, and haloperidol. (**c**) Diagram of gene ontology enrichment analysis. Rich factor: the ratio of the number of target genes divided by the number of all genes in each term Padj: *p* value with multiple testing corrections.

We dose-dependently selected caffeine as a well-known neuronally effective substance. Valproic acid was selected as a representative strong neuronal substance. Furthermore, we analyzed the effects of haloperidol on global gene expression, and next-generation RNA sequencing revealed that each treatment had an expression characteristic (Fig. 3a). We observed both upregulation and downregulation of genes in all comparisons compared with the control (Fig. 3b). The gene lists with expression changes over 5 log2-fold in each comparison are shown in Supplementary Table 1a, b. Please note that there was no obvious difference in the number of changed genes between the low- and high-caffeine treatments. However, through statistical gene ontology (GO) enrichment analysis, we found that the categories of “passive transmembrane transport activity” and “channel activity” were commonly changed in all positive groups but not in the low-dose caffeine group (Fig. 3c).

### Methodological improvement in machine learning (ML) of waveforms

**Figure 4:**
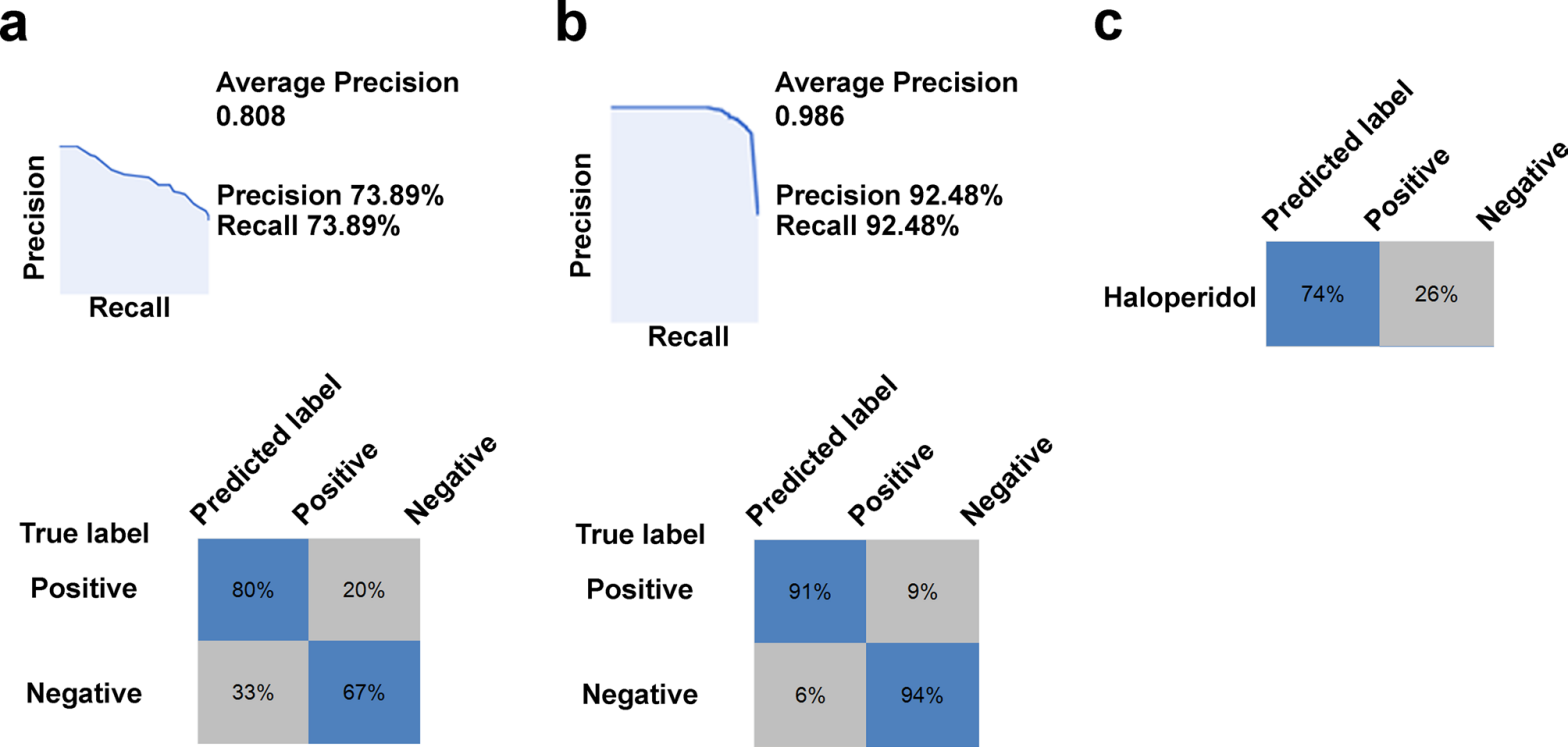
The effects of the new machine learning (ML) methodology. (**a**) MLs were performed using a single-waveform image for training. (**b**) MLs were performed using composite images (nine waveforms in one image) for education. (**c**) Prediction of haloperidol as a new substance belongs to the neuronal activity-positive group.

We first obtained the collections of single 1162 or 1034 waveforms selected from the waveform stocks obtained from the negative or positive group (except waveforms of haloperidol treatment). Next, we demonstrated each ML by each single 1970 waveform randomly selected from the waveform stocks. To evaluate the developed MLM, we tested the precision and recall using 226 new waveforms randomly selected from the stock. As a result, we obtained approximately 81% average precision (Fig. 4a). Next, to ensure that ML focuses on meaningful differences without human data selection, we combined nine randomly selected (image-reuses are not considered) waveforms from each group into one composite image and then demonstrated ML using 1162 composite images. As a result, we obtained approximately 99% average precision (Fig. 4b). Furthermore, to confirm the potential applicability of this MLM (named MLM-1), we challenged noneducated haloperidol -treated calcium waveforms and found that 74% of the waveforms could be segregated into the positive group (Fig. 4c). Further to investigate the width of the applicability of the MLM-1, we treated docosahexaenoic acid (DHA) and gamma-aminobutyric acid (GABA) to newly prepared iNSCs from the frozen stock and obtained their calcium waveforms. The MLM-1 succeeded in segregating GABA into the positive group, but failed to make the correct prediction for DHA (data not shown). To investigate cell-lot dependency of MLMs, we conducted a cross-prediction test by challenging previously obtained waveforms from different lots of iNSCs derived from the same master stocks and found MLM-1 failed to correctly segregate some of the waveforms (data not shown). Next, we newly cultured iNSCs from master stocks, obtained all substances and dose-treated waveforms, and conducted a new ML with composite images. This new MLM (named MLM-2) achieved approximately 89% average precision and succeeded to segregate noneducated haloperidol and DHA-treated waveforms into the positive group with 85% and 59% accuracies, respectively; however, it failed in the case of GABA (segregated only 23% of the waveforms into the positive group) (Supplementary Fig. 4). Further to investigate whether ML discriminates against the recognition of general parameters, we performed statistical analyses of waveforms comparing negative and positive groups regarding spike frequency and dynamic range. We could not find any significant differences in these parameters (Supplementary Fig. 5a, b). Furthermore, to obtain mechanistic insight into the high detection ability of our new ML method, we performed ML using intensively selected weak-signal waveforms. The developed MLM-3 developed with single 703 waveforms did not show superiority over the MLM developed using nonselected waveforms (Supplementary Fig. 6a). In contrast, MLM-3 developed using 703 composite images with the selected weak-signal waveform resulted in better average precisions than the case of MLM-1 developed by composite images using nonselected waveforms (Supplementary Fig. 6b). To further characterize our new ML method, we performed MLs using the same number of waveforms in both single and composite waveform images. We obtained similar average precisions of 0.829 and 0.809, respectively (Supplementary Fig. 7).

## Discussion

This report confirmed calcium signal transduction activity via P2X7 expressed in human iNSCs. The physiological significance of P2X7 in NSCs in vivo is currently not sufficiently characterized; however, our observations show that iNSCs treated with ATP showed accelerated growth, and treatment with Brilliant Blue G harmed this growth (data not shown), similar to what has been reported in tumor cells. Altogether, NSCs may control their growth by sensing pathological conditions^28^. In contrast, mature neurons undergo apoptosis when P2X7 is stimulated^29^. These opposite responses in NSCs, tumor cells, and mature neurons might be hypothetically explained by the evolutionarily retained ability of cell-based repair mechanisms such as brain regeneration seen in lower animals^30^. We found that the majority of iNSCs respond to extracellular ATP via P2X7. In addition to P2X7 expression, we suggested that iNSCs also express P2Y receptors via the observation of intracellular calcium waves when the P2X7-mediated calcium sparks were blocked by the P2X7-specific antagonist Brilliant Blue G^21–24^.

iNSCs are not homogeneous populations. Adenosine triphosphate (ATP) treatment results in mixed responses of random calcium-sparks and waves with various dynamic ranges. Previous studies have applied the ML method to analyze waveforms as digitized images^31^ or as one-dimensional time-series data^32^. We hypothesized that ML using raw waveform images could be more effective for handling the high-variety waveform data of calcium sparks. However, the first ML trial that used single waveforms showed unsatisfactory results. We hypothesized that the possible reason for this failure is that ML could be highly affected by the wide deviations of the signal dynamic ranges of waveform data and decided to try ML using composite images with nine waveforms into one aiming to dilute the effect of such images. Comparing the above two ML methodologies, we found that the latter method can achieve far better prediction precision, suggesting that our new ML strategy might have an advantage when using highly variable waveform signals.

ML has been successfully applied to imaging domains, including in the medical field, to aid in diagnosis by detecting cancerous tissues in image data and distinguishing subtle changes. In most applications in visual domains, humans can attest to the predictions demonstrated by the MLM. In contrast, calcium sparks detected in iNSCs cannot be classified by humans using classical parameters, including frequency or dynamic range, suggesting that our MLM can recognize unidentified features of the waveforms. To investigate this mechanism, we performed ML using only the selected weak-signal waveforms. We found that only the MLM developed using composite weak-signal waveforms resulted in unexpectedly better average precisions than the model developed using composite images including randomly selected waveforms. This could be because ML can focus on essential differences when the dynamic range deviations in the learning data are reduced. Please note that even using selected image MLMs developed by single waveforms showed inferior prediction ability, highlighting the robust superiority of our “waveform composite method.” Finally, we confirmed that similar prediction abilities of MLMs were obtained when using the same numbers of waveforms for MLs, suggesting that the reason why our new ML method can extract meaningful differences in waveforms could be reinforcement through effective repetitive learning using the reuse of waveforms. In contrast to the above merits, we found that the prediction accuracy of the “waveform composite method” is cell-lot dependent, meaning that learning waveforms and testing substance-treated waveforms should be obtained using the same iNSCs. Because even when we used the iNSCs derived from the same master-frozen stocks twice, the developed MLM using one cultured iNSC was not effective for the prediction of the different cell-lot-derived waveforms. This might be because the subtle differences in the iNSCs might affect ML. To minimize the lot-to-lot differences in iNSCs, the usage of an automated culture machine could be a solution^33^. Furthermore, our ML method partially succeeded in the correct segregation of noneducated substances, suggesting ML with larger waveform data sets would be required for more powerful predictability for noneducated substances.

In a similar example, ML has superior data discrimination compared to humans. For example, ML models have been able to detect ventricular premature complexes during sinus rhythm, not during arrhythmia, and it is difficult for even highly trained medical doctors to predict ventricular premature complexes during normal rhythms^32^. The authors discussed a possible mechanism by which MLM can detect subtle ECG structural changes that underline ventricular premature complexes. In the above study, Chang et al*.* performed ML by converting one-dimensional time-series data from two-dimensional images. To our knowledge, our study is the first to report the development of a highly accurate MLM to analyze subtle unidentified alterations developed with raw waveform images with a high degree of variability.

We confirmed that pretreatment had a characteristic effect on the global mRNA expression profile. However, the affected gene expression was very low, except for valproic acid. Surprisingly, our MLM can distinguish the cellular calcium response features based on a few such genetic changes. Therefore, we carefully checked the Gene Ontology (GO)-based categorization of the gene expression changes and found that all positive group members showed common gene expression changes related to “passive transmembrane transport activity” and “channel activity,” suggesting that such changes in transmembrane activities might be the mechanistic bases of the dose-dependent neuronal effects of the tested compound that can be detected by our MLM. The above result also emphasizes that the MLM developed by our new ML method can be a powerful tool to reveal the hidden internal alterations of iNSCs by neuronal effective substances.

## Materials and methods

### Human-induced pluripotent stem cells (hiPSCs)

The human-induced pluripotent stem cell Line 253G1 was obtained from the Laboratory for Pluripotent Cell Studies, RIKEN BioResource Research Center, Tsukuba City, Ibaragi, Japan.

### Human iPSC maintenance

The hiPSCs were maintained in 10 cm plastic plates (Corning Inc., NY, USA) coated with 0.5 μg/cm^2^ iMatrix511 Silk (Nippi Inc., Tokyo, Japan) with StemFit^®^ AK02N (Ajinomoto, Tokyo, Japan) as the culture medium under a controlled atmosphere at 37 °C, 5% CO2, and > 95% humidity. The cells that reached 70–90% confluency were washed three times with phosphate-buffered saline without calcium (PBS(-); Nacalai Tesque Inc., Kyoto, Japan) and treated with 3 ml of TripLE™ express enzyme (Thermo Fisher Scientific, Waltham, MA, USA) supplemented with 10 μM of the Rho kinase inhibitor Y-27632 (Sellec Inc., Tokyo, Japan) for 15 min at 37 °C. Detachment and dispersion into single cells were performed by pipetting using 10 ml pipettes with the addition of 5 ml of PBS(-) supplemented with 0.15% bovine serum albumin fraction V (BSA: Fuji Film Wako Chemical Inc., Miyazaki, Japan). The cells were collected in a 15 ml tube and centrifuged at 800 rpm (115× *g*) for 5 min for deposition. The supernatant was then aspirated, and the cell pellet was dispersed into single cells using 10 ml Stemfit supplemented with 10 μM Y-27632 by pipetting.

### Differentiation of hiPSC-derived neuronal stem cells (iNSCs)

To initiate differentiation (Day 1), undifferentiated iPSCs were seeded at a density of 25,000 cells/cm^2^ in 1/100-diluted Matrigel^®^ (Corning Inc.) -coated 10 cm dish with 10 ml of Neuro ectodermal inducing medium consisting of Dulbecco's modified Eagle medium (DMEM)/F12 (Fuji Film Wako Chemical Inc.) and Neurobasal medium (1:1; Thermo Fisher Scientific) supplemented with 0.1 mM nonessential amino acids, 1 mM GlutaMAX^®^ (Thermo Fisher Scientific) with 10 μM TGF-beta receptor inhibitor (SB431542), 10 μM BMP signal inhibitor (LDN-193189), and 10 μM Y-27632. Media changes were performed on Day 3. On differentiation Day 5, the medium was changed to Neuro-ectodermal-inducing medium supplemented with 10% Knockout serum replacement (Thermo Fisher Scientific), 50 μg/mL ascorbic acid 2-phosphate (Merck), and 10 ng/ml bFGF (Nacalai Tesque Inc.). Media changes were performed on Days 7 and 8. On differentiation Day 9, the cells were passaged to split into three 10 cm dishes with 10 ml of iNSC-expansion medium consisting of DMEM/F12 and neurobasal medium 1:1 supplemented with 0.1 mM nonessential amino acids, 1 mM GlutaMAX^®^, 1% N-2 supplement (Thermo Fisher Scientific), 1% B-27 (Thermo Fisher Scientific) supplement, 10% Knockout serum replacement, and 50 ng/ml bFGF. The media were changed daily. The cells that reached 100% confluence were passaged again for expansion. The resulting cells were cryopreserved using CELLBANKER-1 (Zenogen Pharma Co., Ltd., Fukushima, Japan).

### Treatment of iNSCs and calcium imaging

The cryopreserved iNSCs were dissolved and seeded in Matrigel^®^-coated 10 cm dishes with 10 ml of the iNSC-expansion medium and cultured in an incubator until they reached confluence. After washing once with 10 ml PBS(-), the cells were detached and dissociated using 4 ml of TrypLE Express at 37 °C for three minutes. The cells were centrifuged into a pellet and then dissociated using iNSC-expansion medium without bFGF and several pipetting steps. The 1/20 fraction of the harvested cells was seeded into a Matrigel-coated glass-bottom dish (Cell Imaging dish 170 μm, 35 mm, Eppendorf, Hamburg, Germany). The next day, the medium was replaced with 500 μl of iNSC-expansion medium without bFGF containing the test substances listed in Table 1. The cells were then treated for 12–16 h in an incubator. Then, the medium was replaced with 500 μl of prewarmed iNSC expansion medium without bFGF containing Fluo4-AM (Biotium Inc., Fremont, CA, USA) and incubated at 37 °C for 30 min for dye loading. The cells were washed with 500 μl of prewarmed iNSC-expansion medium without bFGF, and then the medium was changed with 500 μl of prewarmed iNSC-expansion medium without bFGF containing 20 μM ATP (Nacalai Tesque Inc.). After ATP treatment, cellular fluorescence rapidly increased and gradually decreased in approximately 200 s (Supplementary Fig. 8). Therefore, the recording of the calcium sparks was started 200 s after the addition of ATP. Thereafter, we sequentially recorded for 5 min in at least three randomly selected fields of view. Time-lapse images (512 × 512 pixels) were taken every 1 s with a 100 ms exposure time (Eclipse Ti2, Nikon Instruments, Tokyo, Japan). The obtained images were exported as video in audio video interleave (AVI) format using NIS Elements software (Nikon Instruments). To investigate the effect of P2X7 inhibition on ATP-induced calcium sparks, 20 μM BBG, 20 μM JNJ-47965567, or 10 μM AZD9056 was added to the medium before ATP challenge.

### Time-lapse image processing and machine learning

The acquired calcium imaging videos were contrast-enhanced, smoothed with ImageJ software, and converted to grayscale multitiff files. For the converted data, the MATLAB toolbox published by Romano et al. was used to trace changes in fluorescence intensity and convert them into waveform data^34^. Regions of interest (ROIs) were set using hexagonal segmentation with a diameter of 20 μm. The baseline fluorescence noise scale was estimated by fitting it into a Gaussian model. Spike-containing ROIs were selected using the toolbox parameter “smooth slow dynamic” threshold mode. Waveform data were created in the Joint Photographic Experts Group format. Waveform data were used for ML, either one waveform as one data point or one data point having a combination of nine randomly selected waveform images. MLs were performed using AutoML Vision on the Google Could Platform (https://cloud.google.com/automl). AutoML Vision randomly split 90% of the total for training and validation and 10% for testing. Testing of the intragroup data and the ML model (MLM) unexperienced new data was performed by inputting each waveform into the MLM.

### Gene expression profiling by RNA sequencing

iNSCs were treated with none, with low-dose caffeine as the negative group, and with high-dose caffeine, valproic acid, and haloperidol as the positive group, as described in the calcium imaging section. Total RNA was extracted using Isogen and treated with RNase-free DNase (QIAGEN, Venlo, Netherlands). Library construction and sequencing were performed by Novogene Co. Ltd. (Beijing, China). Briefly, the enriched mRNA was fragmented randomly, followed by cDNA synthesis using an mRNA template and random hexamer primers. After second-strand synthesis, sequencing adaptor ligation was performed. Completed double-stranded cDNA libraries were developed using size selection and PCR enrichment. RNA libraries were sequenced on a HiSeq sequencer (Illumina). Bioinformatic analyses were performed by Novogene Co., Ltd.

### Immunohistochemistry

The cells were fixed in 4% paraformaldehyde for 5 min at 25 °C. After that, the cells were washed twice with Tris-buffered saline containing 0.2% Tween-20 (TBS-T) and treated with a blocking solution (Nacalai Tesque) for 30 min at 25 °C. The primary antibody-containing blocking agent was added to the cells and incubated overnight at 4 °C with paraffin sealing to prevent evaporation. The cells were washed three times with TBS-T and immersed in the secondary antibody-containing blocking agent for 1 h at room temperature. After three washes, the fluorescence signals were observed using a fluorescence microscope (Nikon Instruments, Tokyo, Japan). The primary and secondary antibodies used are listed in Supplementary Table 2.

### Western blotting

NSCs were washed once with PBS(-) and lysed using RIPA buffer (Nacalai Tesque Inc.) with a protease inhibitor cocktail (#08714, Nacalai Tesque Inc.). We performed sodium dodecyl sulfate‒polyacrylamide gel electrophoresis using 4–12% Bolt™ Bis-Tris and a 1.0 mm Mini Protein Gel system. The proteins were transferred onto a polyvinylidene fluoride membrane (#1214726, GVS, S.p.A. Rome, Italy) and analyzed by western blotting. The primary and secondary antibodies used are listed in Supplementary Table 1. The luminescent signal was obtained using a horseradish peroxidase substrate (Amersham™ ECL™ Prime, Cytiva, Tokyo, Japan) and captured using Fusion Solo-S (Vilber, Collégien, France). As the internal control, we detected beta-Actin protein using the same membrane reprobed after treatment with stripping solution (Fuji Film Wako Chemical Inc.).

### Quantitative polymerase chain reaction (qPCR) analysis

Total RNA was extracted from the cells using ISOGEN (Nippon Gene, Tokyo, Japan) according to the manufacturer’s instructions. Reverse transcription of 500 ng total RNA was performed using a Versa cDNA synthesis kit (Thermo Fisher Scientific). Quantitative polymerase chain reaction was performed using Thunderbird^®^ Next SYBR^®^ qPCR Mix (Toyobo, Co. Ltd., Osaka, Japan) with gene-specific primer sets for nestin and p2 × 7 mRNA. All experiments were performed using three independent samples. All gene expression levels were normalized to internal ribosomal protein S18 RNA expression levels. The primer pair sequences are listed in Supplementary Table 3.

### Statistical analysis

Statistical analyses were performed by unpaired Student’s *t* test using Microsoft Excel software. Statistical significance was set at *P* < 0.05.

### Supplementary Information

Supplementary Information 1.

Supplementary Video 1.

Supplementary Video 2.

## Data Availability

Raw mRNA sequencing data are available in the DNA Data Bank of Japan (DDBJ, Bio Project Accession number: PRJDB15077). The other original contributions presented in this study are included in the article and supplementary materials, and further inquiries can be directed to the corresponding author.
