# Supplementary material for: Machine learning discriminates P2X7-mediated intracellular calcium sparks in human-induced pluripotent stem cell-derived neural stem cells
Source: Sci Rep. 2023 Aug 4;13:12673. doi: 10.1038/s41598-023-39846-4 (PMC10403609; doi:10.1038/s41598-023-39846-4)

Supplementary materials

Machine learning discriminates P2X7 mediated intracellular calcium sparks of Human-induced pluripotent stem cell-derived neural stem cells

Yuki Hanafusa[1,2#], Akira Shiraishi[3], and Fumiyuki Hattori[1*#]

[#]Authors equally contributed

[1] Innovative Regenerative Medicine, Graduate School of Medicine, Kansai Medical University

[2] Group Quality Assurance Division, Safety Science Institute, Suntory Holdings Ltd.

[3] Division of Integrative Biomolecular Function, Bioorganic Research Institute, Suntory Foundation for Life Sciences.

***Correspondence**: Fumiyuki Hattori

hattorif@hirakata.kmu.ac.jp

Supplementary Tables

Supplementary Table 1a Five log2-fold increased gene lists in each comparison

Caf-H vs None		Caf-L vs None		Valproic acid vs None		Haloperidol vs None	
log2Fold Change	GeneName	log2Fold Change	GeneName	log2Fold Change	GeneName	log2Fold Change	GeneName
7.1	AOAH	6.7	AC098851.1	10.1	BARX1	6.7	ZNF295-AS1
6.5	PSMB8-AS1	6.3	PCNPP1	10.0	LARGE2	6.5	AC090061.1
6.5	AC098851.1	6.3	AC013472.3	9.5	LGI3	6.5	AC240565.2

6.3	NEUROG3	6.3	MIR1262	9.4	GABRD	6.4	PSMB8-AS1
6.3	AC138473.1	6.2	AL451050.2	9.4	PHOSPHO1	6.4	AC109583.2
6.3	AL121906.1	6.0	AP002851.1	9.3	KIF12	6.4	AC097467.3
6.3	AC091614.2	6.0	AC138473.1	9.3	VDR	6.2	MMD2
6.1	DNAH9	6.0	RDH10-AS1	9.2	MUC3A	6.2	ADAMTSL4-AS1
6.1	FXYD2	6.0	AC004801.6	9.1	CYP2S1	6.2	RPL5P18
6.1	AL049795.1	6.0	AC022613.2	9.1	ESX1	6.1	FXYD2
6.1	LINC01776	6.0	AC091946.2	8.9	SPINK2	6.1	AC008494.3
6.1	AC090061.1	5.8	AOAH	8.8	IRX4	6.1	A2ML1
6.1	AP001793.1	5.8	AC022395.1	8.8	IRF6	6.1	RNU1-16P
6.1	AL451050.2	5.8	FXYD2	8.7	HOXD9	6.1	AP001099.1
6.0	C16orf46	5.8	ZNF295-AS1	8.6	L1TD1	5.9	AC098851.1
6.0	RPL26P6	5.8	TBX2	8.5	TNFAIP8L3	5.9	AL451050.2
6.0	AC109462.2	5.8	AC006466.1	8.5	HS3ST6	5.9	AC022395.1
5.8	ALDOB	5.8	PRL	8.5	CYP1A1	5.9	AC004801.6
5.8	PLD4	5.8	AC245595.1	8.4	GPR4	5.9	AP001793.1
5.8	NOXO1	5.8	BGLAP	8.4	SMIM24	5.9	AL359541.1
5.8	METTL11B	5.8	GTF2IP5	8.2	NEUROG3	5.9	AC012354.1
5.8	HNRNPA1P34	5.8	AC068896.1	8.2	RAX	5.9	KCNMB2-AS1
5.8	AC080125.1	5.8	ADAMTS7P	8.2	TCF24	5.9	AC091152.2

			1				
5.8	AL161452.1	5.8	AC109449.1	8.1	FLJ42969	5.9	AC011471.4
5.8	AC092042.1	5.8	AC211486.3	8.1	FOXL2NB	5.9	C2orf91
5.8	AL354726.1	5.8	C12orf71	8.1	ELOVL3	5.9	VIL1
5.8	OSMR-AS1	5.6	LGI3	8.0	ANO3	5.9	AF196972.1
5.8	AC093535.1	5.6	GABRD	8.0	WNT6	5.9	LINC01013
5.8	SMIM6	5.6	ADAMTSL4-AS1	8.0	MYOD1	5.7	RDH10-AS1
5.8	AL445483.1	5.6	AC109583.2	8.0	LANCL3	5.7	AC023906.4
5.8	AC010320.4	5.6	RPL26P6	8.0	TBXT	5.7	DDX39B-AS1
5.8	AL031123.3	5.6	KCNMB2-AS1	8.0	ASPG	5.7	AGGF1P2
5.8	AC091057.5	5.6	CA9	7.9	SHISA8	5.7	APOBEC3D
5.6	TRPC5	5.6	MIR132	7.9	LINC01415	5.7	AC009065.5
5.6	CA9	5.6	DNLZ	7.9	FOXA3	5.7	CBY3
5.6	UROC1	5.6	AC023157.2	7.9	GATA4	5.7	AC119396.2
5.6	CMTM5	5.6	RF00019	7.8	MT1E	5.7	LINC02362
5.6	LGI3	5.6	AC055874.1	7.8	ALOXE3	5.7	SNORA59A
5.6	AL590139.1	5.6	AP001178.2	7.8	TBX2-AS1	5.7	AL121890.5
5.6	RPL5P18	5.6	LRIT1	7.8	C8orf34	5.7	LINC01836
5.6	GAPDHP20	5.6	ITIH2	7.8	GMNC	5.7	HEATR9
5.6	ELOCP21	5.6	AC005277.2	7.7	PLD4	5.7	C1orf232
5.6	AC005013.1	5.6	RF02272	7.7	TPBGL	5.7	FAM192BP

5.6	CICP27	5.3	LPAR3	7.7	PRKCH	5.7	ANGPTL6
5.6	DDX39B-AS 1	5.3	KCNJ3	7.7	MYZAP	5.7	AC009093.1
5.6	AL512622.1	5.3	AC090061.1	7.6	BEX5	5.5	L1TD1
5.6	AC117944.1	5.3	PSMB8-AS 1	7.6	GRB7	5.5	AP002851.1
5.6	AC104623.1	5.3	PCDH8P1	7.6	LPAR3	5.5	FOXE1
5.6	LINC01119	5.3	RPL5P18	7.6	GABRB1	5.5	PROCR
5.6	RF00019	5.3	AL121906.1	7.6	PRSS16	5.5	AL049795.1
5.6	AC023906.4	5.3	KCNG3	7.6	HAND1	5.5	ANKRD20A1
5.6	THCAT158	5.3	RNU6-45P	7.6	HOTAIRM1	5.5	RPL26P6
5.6	AC010522.1	5.3	ANKRD33	7.5	AP003774.1	5.5	AL121906.1
5.6	AC026150.3	5.3	STK31	7.5	PCDH8P1	5.5	AC006466.1
5.6	HIST2H4B	5.3	AC004771.1	7.5	SMIM32	5.5	DNLZ
5.6	AL589739.1	5.3	AC055855.1	7.4	PTHLH	5.5	AC055855.1
5.6	AC109583.2	5.3	TMEM235	7.4	DQX1	5.5	AC011369.2
5.6	AC011369.2	5.3	AC137723.1	7.4	RAET1G	5.5	TMEM184A
5.6	AC240565.2	5.3	AC117382.2	7.4	FOXI3	5.5	AL008628.1
5.6	AC006581.2	5.3	RNU12-2P	7.4	VAX1	5.5	AL162311.3
5.6	AP002851.1	5.3	AC021755.2	7.4	VENTX	5.5	AC068025.1
5.4	AFP	5.3	AC254562.2	7.4	AL022344.2	5.5	OPA1-AS1
5.4	PTHLH	5.3	MIR579	7.4	AC040174.1	5.5	SUMO2P6
5.4	PROCR	5.3	NPR3	7.4	NKX2-6	5.5	LINC01103

5.4	ANO3	5.3	AC090772.1	7.4	GATA6	5.5	AC005740.3
5.4	MMD2	5.3	AC245100.2	7.3	KCNJ3	5.5	LINC02244
5.4	TTC6	5.3	PDC	7.3	FOXE1	5.5	AC091729.1
5.4	SSTR1	5.3	AC026412.2	7.3	CA7	5.5	AC022819.1
5.4	DUOX2	5.3	AL135744.1	7.3	TBX3	5.5	AL021396.1
5.4	LANCL3	5.3	AC104365.2	7.3	CLDN6	5.5	AL031658.2
5.4	FRMPD2	5.3	AC114284.1	7.2	ADRB2	5.5	AC025031.1
5.4	P2RY6	5.3	AC005740.1	7.2	NCCRP1	5.5	RPL41P2
5.4	OR52N4	5.3	AC092849.1	7.2	UCP1	5.5	MEFV
5.4	MIXL1	5.3	CHRNB3	7.2	CYP24A1	5.5	GUSBP11
5.4	RF00019	5.3	AL359922.3	7.2	HOXA13	5.5	LINC01964
5.4	ADAMTSL4-AS1	5.3	PRSS8	7.2	BSPRY	5.5	AC010680.2
5.4	CBY3	5.3	AC004947.1	7.2	ADGRG5	5.5	HNRNPA1P14
5.4	C11orf91	5.3	GLIPR1L2	7.2	MCOLN2	5.5	RN7SL382P
5.4	OPA1-AS1	5.3	AL359399.1	7.2	AMBN	5.5	RF00019
5.4	AC012354.1	5.3	LINC01191	7.2	SLC15A1	5.5	AC011509.1
5.4	LEMD1-AS1	5.3	RF00019	7.2	FGF4	5.5	FEN1P1
5.4	AL359541.1	5.3	AC012676.4	7.2	PCP4L1	5.5	NME1-NME2
5.4	LTA	5.3	AL049697.3	7.1	AF111167.2	5.2	TNFAIP8L3
5.4	AL158835.1	5.3	TCP1P1	7.1	AC004264.2	5.2	LANCL3

5.4	AC007036.2	5.3	ZNF883	7.1	RAC2	5.2	LPAR3
5.4	RPS3P2	5.3	AC022968.1	7.0	NKX2-3	5.2	PTHLH
5.4	AC245595.1	5.3	AC007786.1	7.0	A4GALT	5.2	DQX1
5.4	AC092814.1	5.3	BTF3L4P1	6.9	PROCR	5.2	AC091614.2
5.4	GTF3C2-AS1	5.3	AC017002.3	6.9	FAM86GP	5.2	ANKRD33
5.4	DCTN1-AS1	5.3	MTND2P21	6.9	LINC01220	5.2	STK31
5.4	AC002368.1	5.3	AC011726.3	6.9	AC097639.1	5.2	METTL11B
5.4	KCNMB2-AS1	5.3	AC124944.1	6.9	LINC02600	5.2	GTF3C2-AS1
5.4	PPATP1	5.3	AC005180.1	6.9	HLA-F	5.2	AC005013.1
5.4	FLNC-AS1	5.3	RNU6-608P	6.9	AC147067.1	5.2	LINC01119
5.4	APOBEC3D	5.3	PSORS1C2	6.9	DSP	5.2	AL033384.2
5.4	RNU7-79P	5.3	XRCC6P4	6.9	ESRP1	5.2	TMEM235
5.4	EID3	5.3	RPL34P20	6.9	HMX2	5.2	AC010487.2
5.4	AC023157.2	5.0	CYP1A1	6.8	AL512622.1	5.2	PFN1P3
5.4	OR5BK1P	5.0	FLJ42969	6.8	KCNE1B	5.2	SIX3-AS1
5.4	PCNPP1	5.0	TPBGL	6.8	PRSS3	5.2	AC097478.1
5.4	HIF1A-AS2	5.0	PTHLH	6.8	SNX18P3	5.2	AC103952.1
5.4	AC009654.1	5.0	GABRB1	6.8	AC008556.1	5.2	AC006581.2
5.4	AF111167.2	5.0	VAX1	6.8	AL109809.1	5.2	AC114316.2
5.4	AC135782.3	5.0	AL512622.1	6.8	GBX1	5.2	GAPDHP62
5.4	AC117382.2	5.0	AC091614.2	6.8	BTNL9	5.2	AC007229.1
5.4	TCF24	5.0	AC023906.4	6.8	LINC00605	5.2	SNORD46

5.4	MIR132	5.0	AP001793.1	6.8	C2CD4B	5.2	AL133297.1
5.4	TBX2-AS1	5.0	AL049795.1	6.7	AOAH	5.2	ABCG8
5.4	AC011481.2	5.0	LINC01776	6.7	MMD2	5.2	RF00019
5.4	AC012309.1	5.0	ANKRD20A1	6.7	MIXL1	5.2	AC132008.1
5.4	AF038458.2	5.0	DDX39B-AS1	6.7	KCNG3	5.2	KRT8P26
5.4	AC010327.2	5.0	AC012354.1	6.7	TBX2	5.2	VWA8-AS1
5.4	AC007229.1	5.0	C12orf56	6.7	C12orf56	5.2	AC010999.2
5.4	AC119396.2	5.0	AC009654.1	6.7	AL353746.1	5.2	SNORA22
5.4	AC239600.1	5.0	METTL11B	6.7	AC113414.1	5.2	AC104964.1
5.4	AC008494.3	5.0	GTF3C2-AS1	6.7	LINC02495	5.2	ITIH2
5.4	AC080013.6	5.0	AC080013.6	6.7	TBX20	5.2	PRMT5-AS1
5.4	AC027271.1	5.0	C16orf46	6.7	CERNA1	5.2	AL845552.2
5.4	MIR8064	5.0	LINC01119	6.7	SNCB	5.2	RF02271
5.4	AL121583.1	5.0	AL355601.1	6.6	AC022395.1	5.2	AL020997.1
5.4	AC244093.5	5.0	APOBEC3D	6.6	ANKRD20A1	5.2	AC082651.3
5.4	AL158212.4	5.0	AC080125.1	6.6	AL137847.1	5.2	AC012485.1
5.4	AC040160.2	5.0	AC010487.2	6.6	AC020934.1	5.2	AC116347.1
5.4	AC036214.4	5.0	SIX3-AS1	6.6	DLX6	5.2	AC092032.1
5.4	AC012673.1	5.0	AL844908.2	6.6	AL031658.1	5.2	SPIN4-AS1
5.2	TLDC2	5.0	PADI1	6.4	AC078785.2	5.2	LINC02378

5.2	ACP5	5.0	AC114316.2	6.4	TNFSF10	5.2	RPL12P7
5.2	EPHX3	5.0	NOXO1	6.4	FOXG1	5.2	NARF-AS1
5.2	EBI3	5.0	AC068025.1	6.4	LINC01096	5.2	SRGAP2-AS1
5.2	SLC6A4	5.0	EPHX3	6.4	DUX4L9	5.2	AC063944.3
5.2	CFHR3	5.0	LTA	6.4	TSLP	5.2	AC104806.2
5.2	AVPR2	5.0	LINC01801	6.4	HOXC13	5.2	FAM87A
5.2	DQX1	5.0	SKINT1L	6.4	PAX9	5.2	AL138752.1
5.2	PDZD9	5.0	AC005740.3	6.4	HOXB7	5.2	MIR4477B
5.2	KCNJ3	5.0	HS3ST4	6.4	VSTM2B	5.2	AL353583.1
5.2	DPPA2	5.0	AC005775.1	6.4	DHRS2	5.2	AC006116.6
5.2	TMEM184A	5.0	PPP1R8P1	6.3	AL451050.2	5.2	SLC22A13
5.2	MT1E	5.0	CYP4F27P	6.3	AC022613.2	5.2	AC133473.1
5.2	AC138356.1	5.0	PWRN1	6.3	AL161452.1	5.2	SPDYE20P
5.2	SPATA45	5.0	AL031658.2	6.3	RNU6-45P	5.2	RN7SL521P
5.2	GABRD	5.0	EBI3	6.3	C11orf91	5.2	POF1B
5.2	AC079610.1	5.0	AC119674.1	6.3	HUS1B	5.2	RNU6-1331P
5.2	CNGA1	5.0	AC073410.1	6.3	FTCD	5.2	AP001271.1
5.2	SNORD73B	5.0	CPA1	6.3	AC026250.1	5.2	AC027682.5
5.2	RF00019	5.0	LINC02378	6.3	AC079296.1	5.2	RF00019
5.2	NXPE2	5.0	RPL12P7	6.3	TBX4	5.2	AC019080.3
5.2	SNORA22	5.0	RNU2-69P	6.3	ATP12A	5.2	AC110994.2
5.2	SNORA46	5.0	NKG7	6.3	AC129492.4	5.2	AC092656.1
5.2	MIR593	5.0	GUSBP11	6.3	TMEM92	5.2	YBX1P4

5.2	MIR106B	5.0	AC053503.1	6.3	SLC30A2	5.2	ZP1
5.2	TRDJ2	5.0	GAPDHP42	6.3	LINC02522	5.2	FABP5P2
5.2	AL807752.1	5.0	PRSS3P4	6.3	HOXA1	5.2	AC055876.2
5.2	KRT8P26	5.0	AC098934.3	6.3	PABPC1L2B	5.2	MIR1972-1
5.2	MIR1302-8	5.0	AC133785.1	6.3	C11orf88	5.2	C20orf203
5.2	MIR1285-1	5.0	LINC01821	6.3	AC121338.2	5.2	HIGD1AP16
5.2	NPM1P39	5.0	LMOD3	6.3	CRHR1	5.2	EZR-AS1
5.2	AL596247.1	5.0	F11-AS1	6.2	RTBDN	5.2	ARHGEF5
5.2	AL445490.1	5.0	AC091849.2	6.2	AFAP1L1	5.2	AC239367.3
5.2	AC004771.1	5.0	RPL12P1	6.2	AP002851.1	5.2	RNA5SP282
5.2	AC073987.1	5.0	AP001271.1	6.2	RDH10-AS1	5.2	MANSC4
5.2	RPS6KA2-AS1	5.0	TAS2R64P	6.2	AC023906.4	5.2	LINC02345
5.2	AC231533.1	5.0	AL139142.2	6.2	AC112236.1	5.2	AC100827.5
5.2	AC244453.2	5.0	AC022336.1	6.2	TRPM8	5.2	AC131097.2
5.2	LINC01136	5.0	AL021918.3	6.2	AC008060.3	5.2	AL137918.1
5.2	LINC01103	5.0	RPL21P65	6.2	ATAD3C	5.2	RF00618
5.2	LINC01239	5.0	MYO1A	6.2	SCARNA6	5.2	AC007608.2
5.2	SHISA8	5.0	PCOTH	6.2	AC007375.2	5.2	AC138627.1
5.2	AC097533.1	5.0	LINC00458	6.2	TRIM54	5.2	AC092120.1
5.2	AL136126.1	5.0	GCNT3	6.2	HTR6	5.2	RN7SL146P
5.2	FGF14-IT1	5.0	SCARNA1	6.2	UPK1A-AS1	5.2	MIR769
5.2	LEFTY1	5.0	AL606760.3	6.2	ELFN2	5.2	AC106795.3

5.2	RN7SL395P	5.0	AC092902.1	6.2	CDC20B	5.2	RNU7-84P
5.2	SNRPCP3	5.0	RF00019	6.2	AC025183.1	5.2	RN7SL330P
5.2	TNXA	5.0	AC137894.1	6.2	PDX1	5.2	AC055876.4
5.2	RDH10-AS1	5.0	AL360267.2	6.1	SPAG6	5.2	RN7SKP217
5.2	KLHL2P1	5.0	AC018362.1	6.1	PLPP2	5.2	RF00019
5.2	AC008667.2	5.0	RF00019	6.0	AC004801.6	5.2	AC009152.3
5.2	AC097467.3	5.0	AC022748.2	6.0	ANKRD33	5.2	AP001172.2
5.2	AC083843.1	5.0	ZNF439	6.0	AC009654.1		
5.2	TDGF1P5	5.0	LHB	6.0	AC004771.1		
5.2	AP002812.3	5.0	BX539320.1	6.0	RHOXF1P3		
5.2	AP003049.2	5.0	AC090044.1	6.0	AL355601.1		
5.2	AC087277.2	5.0	TMED7-TICAM2	6.0	RPS6KA2-AS1		
5.2	AP003718.1	5.0	AC008629.2	6.0	AC137723.1		
5.2	AP002770.2	5.0	Z99289.2	6.0	PADI1		
5.2	AC016705.1	5.0	S100G	6.0	RNU6-678P		
5.2	AC022613.2	5.0	AC022868.1	6.0	TRPA1		
5.2	LINC02244	5.0	AC127002.1	6.0	AL356804.1		
5.2	LINC01220	5.0	MRAP	6.0	AC012123.1		
5.2	AC023043.1	5.0	AC105402.2	6.0	AL033527.3		
5.2	TPBGL	5.0	THAP12P2	6.0	TXK		
5.2	AC006030.1	5.0	AC074044.1	6.0	ZMYND15		
5.2	MIR212	5.0	AC006148.1	6.0	AC016907.2		

5.2	SLC25A1P5	5.0	CBX1P1	6.0	M1AP		
5.2	LINC01801	5.0	AC034114.2	6.0	AC004835.1		
5.2	AC011466.1	5.0	HHLA1	6.0	AC022239.1		
5.2	AC008555.2	5.0	ACOT6	6.0	SLC5A5		
5.2	AL035427.1	5.0	AC015819.1	6.0	AC245100.7		
5.2	AC145285.5	5.0	PLEKHA3P 1	6.0	DUX4L26		
5.2	AC010424.2	5.0	AP000317.2	6.0	AL049794.1		
5.2	AC013472.3	5.0	AC006511.5	6.0	AL391845.2		
5.2	AC006023.2	5.0	AC011933.1	6.0	TBX15		
5.2	AC083806.2	5.0	MTCO2P2	6.0	HAGLROS		
5.2	RF00066	5.0	AC136469.1	6.0	MYMX		
5.2	RF00003			6.0	TBX18		
5.2	MIR3648-1			6.0	HOXA4		
5.2	AC004263.1			6.0	BTG4		
5.2	AL136379.1			6.0	KLK1		
5.2	AC110048.2			5.9	SOWAHA		
5.2	AC018926.3			5.9	TMOD1		
5.2	HIST1H2AB			5.9	AC090061.1		
5.2	AC091965.5			5.9	AC138473.1		
5.2	AC100788.2			5.9	AC091614.2		
5.2	ATP6V0CP4			5.9	LINC01776		
5.2	AL358472.6			5.9	AC091152.2		

				5.9	AL033384.2		
				5.9	AP003555.3		
				5.9	AC010487.2		
				5.9	RPL12P25		
				5.9	CRB3		
				5.9	AC243960.2		
				5.9	AC011481.2		
				5.9	RNU12-2P		
				5.9	AC025043.1		
				5.9	RNU4-47P		
				5.9	NPR3		
				5.9	MAT1A		
				5.9	AL021918.4		
				5.9	AC005014.1		
				5.9	FOXI2		
				5.9	AC078778.2		
				5.9	AC090340.1		
				5.9	DNAJC5G		
				5.9	AC008443.3		
				5.9	HLA-DPB2		
				5.9	STK32A-AS1		
				5.9	PABPC1L2A		
				5.9	AC027117.2		

				5.9	OVOL1		
				5.9	LINC01405		
				5.9	ADCY4		
				5.9	AL160313.2		
				5.9	KRT19		
				5.9	EXOC3L2		
				5.9	SNORD88A		
				5.9	ALX3		
				5.9	TDRD10		
				5.9	RXRG		
				5.9	ECEL1P2		
				5.9	FOXD4L3		
				5.9	BBOX1-AS1		
				5.9	TRIM72		
				5.8	NSFP1		
				5.8	USH1G		
				5.8	DLX4		
				5.7	SLC7A10		
				5.7	SUSD3		
				5.7	AC098851.1		
				5.7	PCNPP1		
				5.7	DNAH9		
				5.7	AC093535.1		

				5.7	PRL		
				5.7	AC083806.2		
				5.7	AC100788.2		
				5.7	AC112487.1		
				5.7	NAP1L4P1		
				5.7	MAFA-AS1		
				5.7	AP000640.1		
				5.7	SSTR1		
				5.7	AC061975.1		
				5.7	AL021154.1		
				5.7	PKD1L1		
				5.7	CHRNA2		
				5.7	TRPC6		
				5.7	AL137058.1		
				5.7	AL352977.2		
				5.7	YES1P1		
				5.7	SLCO2A1		
				5.7	SAMD3		
				5.7	AL096816.1		
				5.7	AL031651.2		
				5.7	HORMAD2-AS1		
				5.7	AC016405.2		

				5.7	INSL6		
				5.7	AL592437.1		
				5.7	MISP		
				5.7	CLCNKA		
				5.7	CD1D		
				5.7	RPS3AP14		
				5.7	AC099548.2		
				5.7	NPFFR1		
				5.7	HSPD1P12		
				5.7	OR4D1		
				5.7	TINAGL1		
				5.7	AC019186.1		
				5.7	LINC02084		
				5.7	PSD2-AS1		
				5.7	PENK		
				5.7	LCN15		
				5.7	AC063926.2		
				5.7	RDH12		
				5.7	MT1G		
				5.7	PYY		
				5.7	RNVU1-1		
				5.7	TNFAIP6		
				5.7	OR7E28P		

				5.7	HOXA-AS3		
				5.7	HOXA5		
				5.7	AC004593.1		
				5.7	AL669942.1		
				5.7	AL513128.1		
				5.7	LRCOL1		
				5.7	KCNG4		
				5.7	ALOX15		
				5.7	ARHGEF15		
				5.7	LRRC37A11P		
				5.7	HOXB9		
				5.7	CRLF1		
				5.6	KCNV1		
				5.5	FAM160A1		
				5.5	ADAMTSL4-AS1		
				5.5	RPL5P18		
				5.5	AP001793.1		
				5.5	AL049795.1		
				5.5	STK31		
				5.5	AC080013.6		
				5.5	DNLZ		
				5.5	AC011471.4		

				5.5	SLC6A4		
				5.5	AC097478.1		
				5.5	AC103952.1		
				5.5	AC114316.2		
				5.5	AC027271.1		
				5.5	AC100797.1		
				5.5	FAM95B1		
				5.5	AL592295.3		
				5.5	TBR1		
				5.5	RNU6-720P		
				5.5	LINC01607		
				5.5	MESP2		
				5.5	HS3ST4		
				5.5	AC005775.1		
				5.5	AL139288.1		
				5.5	ZNF806		
				5.5	RF00019		
				5.5	TEX13D		
				5.5	AC105105.4		
				5.5	AC010624.2		
				5.5	IFNE		
				5.5	AL845472.1		
				5.5	CYP4F25P		

				5.5	AL356215.1		
				5.5	AC092384.3		
				5.5	AC008498.1		
				5.5	IL17B		
				5.5	AL139807.1		
				5.5	AC093107.1		
				5.5	SYNDIG1L		
				5.5	ACAN		
				5.5	LCP2		
				5.5	AC073323.1		
				5.5	SH2D1A		
				5.5	AC022784.1		
				5.5	AP001636.3		
				5.5	AC092490.1		
				5.5	OR11M1P		
				5.5	AC079174.1		
				5.5	AL161669.2		
				5.5	AC105339.1		
				5.5	ITM2BP1		
				5.5	AC005523.1		
				5.5	AC008543.4		
				5.5	MPL		
				5.5	CLRN1		

				5.5	S100P		
				5.5	RCC2P7		
				5.5	OTOG		
				5.5	CSRP3		
				5.5	LINC01517		
				5.5	TARDBPP2		
				5.5	ARHGAP40		
				5.5	LINC01814		
				5.5	AC079354.1		
				5.5	AC102953.1		
				5.5	AC104241.2		
				5.5	GLYATL1P4		
				5.5	SENCR		
				5.5	TLX1		
				5.5	CALHM1		
				5.5	MIR3944		
				5.5	AC138466.5		
				5.5	AC007611.1		
				5.5	AC138305.1		
				5.5	TBX21		
				5.5	AC053503.6		
				5.5	AL136360.1		
				5.5	MIR5689HG		

				5.5	HLA-F-AS1		
				5.5	TMEM270		
				5.5	FGF20		
				5.5	SOX17		
				5.5	PTPRD-AS2		
				5.5	OR8B9P		
				5.5	AL138767.3		
				5.5	HOXC10		
				5.5	AC027243.1		
				5.5	MT1A		
				5.5	GCGR		
				5.5	SKOR2		
				5.4	YBX2		
				5.4	WNT16		
				5.4	DGKK		
				5.4	CD40		
				5.4	FNDC1		
				5.3	HMGB1P1		
				5.3	GJA3		
				5.2	PSMB8-AS1		
				5.2	DDX39B-AS1		
				5.2	AL359541.1		
				5.2	MIR132		

				5.2	AC005013.1		
				5.2	AC091946.2		
				5.2	PFN1P3		
				5.2	SIX3-AS1		
				5.2	RN7SL145P		
				5.2	GAPDHP62		
				5.2	AC021755.2		
				5.2	AL133297.1		
				5.2	SKINT1L		
				5.2	LEFTY1		
				5.2	ABCG8		
				5.2	HIST1H2AB		
				5.2	EID3		
				5.2	AC010999.2		
				5.2	AC079610.1		
				5.2	SLC6A1-AS1		
				5.2	SNRPCP3		
				5.2	AC011479.2		
				5.2	HMGB3P9		
				5.2	RNY1P13		
				5.2	AC008663.3		
				5.2	AL139398.1		
				5.2	AC019257.2		

				5.2	LMO7-AS1		
				5.2	AC021439.1		
				5.2	AC108134.2		
				5.2	HIGD1B		
				5.2	NKG7		
				5.2	RNU6-100P		
				5.2	PHF24		
				5.2	AC024257.2		
				5.2	AL355001.1		
				5.2	AC079336.6		
				5.2	MMP28		
				5.2	AC016168.1		
				5.2	PTGS2		
				5.2	ST3GAL5-AS1		
				5.2	AC133785.1		
				5.2	AC122713.1		
				5.2	AF131216.3		
				5.2	AP002986.1		
				5.2	AC009509.4		
				5.2	LINC02418		
				5.2	AL121768.1		
				5.2	AC005921.3		
				5.2	C3		

				5.2	AC008758.6		
				5.2	MIR7856		
				5.2	AC091488.1		
				5.2	SEPHS1P6		
				5.2	MTRNR2L12		
				5.2	LINC01998		
				5.2	CLDN16		
				5.2	DNAH8		
				5.2	EPO		
				5.2	RPA4		
				5.2	AC068020.1		
				5.2	AP002498.1		
				5.2	AC016397.2		
				5.2	PKD1L3		
				5.2	AP000553.6		
				5.2	AC253536.2		
				5.2	LINC01343		
				5.2	GBP5		
				5.2	AC099344.3		
				5.2	FAR2P3		
				5.2	AC092658.1		
				5.2	R3HDM2P1		
				5.2	AL158201.1		

				5.2	KRTAP5-10		
				5.2	AKR1C6P		
				5.2	AL138920.1		
				5.2	WIF1		
				5.2	LINC02288		
				5.2	ISM2		
				5.2	AJ003147.2		
				5.2	AC099494.2		
				5.2	AC015656.1		
				5.2	AL096855.1		
				5.2	G0S2		
				5.2	AC073264.3		
				5.2	AP003721.4		
				5.2	AP006333.1		
				5.2	RF00019		
				5.2	CD163		
				5.2	AC125603.1		
				5.2	AC063926.1		
				5.2	MIR3613		
				5.2	AL442067.2		
				5.2	AC019254.1		
				5.2	STH		
				5.2	AP001010.1		

				5.2	IFI30		
				5.2	CR1L		
				5.2	AL133516.1		
				5.2	ECEL1P1		
				5.2	LINC01988		
				5.2	LINC01391		
				5.2	GABRA4		
				5.2	PFN3		
				5.2	HOXA11		
				5.2	AC073130.1		
				5.2	GPR101		
				5.2	AC100823.1		
				5.2	AL360004.1		
				5.2	OR5A1		
				5.2	PIK3R6		
				5.2	CREB3L3		
				5.2	AC020907.5		
				5.2	GLIS1		
				5.2	TJP3		
				5.1	KLRG2		
				5.1	SCIN		
				5.1	SUSD4		
				5.1	COL12A1		

				5.1	ULBP2		
				5.1	RGL3		
				5.1	TRIM7		
				5.1	CRHR2		

Supplementary Table 1b Five log2-fold decreased gene lists in each comparison

Caf-H vs None		Caf-L vs None		Valproic acid vs None		Haloperidol vs None	
log2FoldChange	GeneName	log2FoldChange	GeneName	log2FoldChange	GeneName	log2FoldChange	GeneName
-6.9	LINC00515	-6.2	RAD51AP2	-7.1	AC009166.2	-6.8	GUSBP3
-6.7	LINC01186	-6.2	RF01241	-7.1	AC091826.2	-6.7	AL139349.1
-6.6	BMS1P12	-6.2	C2orf73	-7.0	GPR179	-6.6	AC026740.2
-6.4	CLDN20	-6.0	AC108047.1	-6.8	AC116407.2	-6.5	IRGM
-6.2	TSPO2	-6.0	AL445187.1	-6.8	AL645504.1	-6.2	AC007823.1
-6.2	AC080188.1	-6.0	TRPV2	-6.6	AL021707.3	-6.0	AC004594.1
-6.1	P2RX2	-6.0	AP000253.1	-6.5	ASIC2	-6.0	AC011603.1

-6.1	PRICKLE2-AS3	-6.0	UNC13C	-6.5	DGCR10	-6.0	AC135050.4
-6.1	LINC00937	-6.0	AC002094.4	-6.5	AC073863.1	-6.0	AC093849.1
-6.1	SUB1P1	-6.0	ARL4AP4	-6.5	GHRH	-6.0	P2RX2
-6.1	DIAPH2-AS1	-5.8	CASTOR1	-6.5	NFE2	-6.0	AC022167.5
-6.1	RN7SL221P	-5.8	AC140912.1	-6.4	UBE2R2-AS1	-6.0	AC087683.1
-6.1	AC087683.1	-5.8	AC104532.2	-6.4	AL512306.2	-6.0	SNORA23
-6.1	AC009878.1	-5.8	UBE2V2P3	-6.4	RNA5SP203	-5.7	AC060834.1
-5.8	C12orf42	-5.8	AC008875.1	-6.4	AL591686.2	-5.7	WTAPP1
-5.8	AC003991.1	-5.8	AC009878.1	-6.4	AC044802.1	-5.7	PDE6G
-5.8	AC006450.1	-5.8	NGB	-6.4	AL133297.2	-5.7	HTR5BP
-5.8	LINC01563	-5.8	RNU6-623P	-6.4	AC013643.3	-5.7	RIPPLY1
-5.8	AL591846.2	-5.8	AC093849.1	-6.4	AC103739.1	-5.7	BNIP3P10
-5.8	NPM1P29	-5.8	SUB1P1	-6.4	ACTG1P3	-5.7	PXT1

-5.8	NOX5	-5.8	RN7SL22<br>1P	-6.2	AC067747.<br>1	-5.7	AC124303<br>.2
-5.8	YBX3P1	-5.8	AC087683<br>.1	-6.2	CACNA2D<br>4	-5.7	AC091891<br>.1
-5.8	AC233992.2	-5.8	SNORA23	-6.2	AC013791.<br>1	-5.7	REV3L-IT<br>1
-5.8	AC245033.3	-5.6	ADGRD2	-6.2	PCDHB19<br>P	-5.7	EEF1A1P<br>12
-5.8	AL356417.3	-5.6	NKX2-5	-6.2	AL021997.<br>3	-5.5	PITX3
-5.6	APOA1	-5.6	AC117500.<br>2	-6.2	CASP4	-5.5	DENND1<br>C
-5.6	PTGER2	-5.6	C4orf45	-6.2	RNF103-C<br>HMP3	-5.5	DPP10-AS<br>1
-5.6	MYBPH	-5.6	AC092535<br>.4	-6.2	AL356740.<br>3	-5.5	RNA5SP1<br>74
-5.6	GJB5	-5.6	IPO4	-6.2	AC080188.<br>1	-5.5	AC009292<br>.1
-5.6	RF00019	-5.6	AC004980<br>.2	-6.2	UNC13C	-5.5	AC025178<br>.1
-5.6	SLC23A3	-5.6	LINC0255<br>4	-6.0	AL035681.<br>1	-5.5	HADHAP<br>2
-5.6	INHBA-AS1	-5.6	AP003419.<br>2	-6.0	DEFB131<br>B	-5.5	ONECUT<br>3

-5.6	FLJ31104	-5.6	WTAPP1	-6.0	TNFSF13B	-5.5	GMFG
-5.6	AL031728.1	-5.6	AL031055.1	-6.0	AL137784.2	-5.5	RF00019
-5.6	AL157834.1	-5.6	MYL12BP1	-6.0	AC006974.2	-5.5	WISP1
-5.6	RPL7L1P3	-5.6	NOX5	-6.0	AC015727.1	-5.5	AL158196.1
-5.6	MED15P5	-5.6	AF131215.7	-6.0	AC020913.3	-5.5	AC243654.3
-5.6	FRY-AS1	-5.6	HNRNPKP2	-6.0	RF00272	-5.5	AC008278.2
-5.6	AP004245.1	-5.6	AC068946.2	-6.0	ABCB10P4	-5.5	EIF3LP3
-5.6	AC104411.1	-5.6	AC016526.3	-6.0	APOBR	-5.5	LINC02177
-5.6	LINC02050	-5.6	PXT1	-6.0	AC234771.4	-5.5	AC146944.3
-5.6	AC010255.1	-5.6	AC124303.2	-6.0	AL390067.1	-5.5	LINC00951
-5.6	AC091180.4	-5.6	RN7SL510P	-6.0	AC107222.1	-5.5	AP001453.2
-5.6	LINC01289	-5.6	PIK3R2	-6.0	AC134312.4	-5.5	AP000755.2

-5.6	AC026191.1	-5.6	AL591846.2	-6.0	AC011603.1	-5.5	AL031283.1
-5.6	AF131215.2	-5.6	EEF1A1P12	-6.0	RF02271	-5.5	LGR6
-5.6	AC124276.1	-5.6	AC003991.1	-6.0	P2RX2	-5.5	RF00019
-5.6	AC048382.1	-5.4	SBK3	-6.0	SNORA23	-5.5	OR7E126P
-5.6	AC243562.3	-5.4	GASAL1	-5.8	AC114490.1	-5.5	UBE2Q2P11
-5.6	AC020978.3	-5.4	TNK2-AS1	-5.8	SMARCA5-AS1	-5.5	AC024337.1
-5.6	DTX2P1-UPK3BP1-PMS2P11	-5.4	CLEC19A	-5.8	AC008277.1	-5.5	AC017104.3
-5.6	LINC01864	-5.4	PTF1A	-5.8	AC019155.1	-5.5	AC103681.2
-5.6	COX6CP16	-5.4	UPP2	-5.8	ZPLD1	-5.5	MAP3K7CL
-5.3	MYBPC2	-5.4	AC009054.2	-5.8	AL391425.1	-5.5	AC019193.2
-5.3	ACR	-5.4	MIR4258	-5.8	RNU7-57P	-5.5	PMS2P7
-5.3	MGP	-5.4	AL356488.1	-5.8	EFCAB14-AS1	-5.5	AC003005.2
-5.3	ABCG2	-5.4	GNRHR2	-5.8	AC015802.	-5.5	GRHL2

			P1		1		
-5.3	OCM	-5.4	AC244197.2	-5.8	RNA5SP69	-5.5	AC010255.1
-5.3	ANGPTL3	-5.4	AC005726.2	-5.8	AC008937.1	-5.5	SLC23A3
-5.3	ERG	-5.4	AC100793.2	-5.8	GLUD1P8	-5.5	RNU2-34P
-5.3	HSD17B13	-5.4	POU5F1P6	-5.8	AL031055.1	-5.5	RPL7L1P3
-5.3	ZNF596	-5.4	NUP210P3	-5.8	AC006974.1	-5.5	FARSA-AS1
-5.3	CCDC63	-5.4	LINC00951	-5.8	NUFIP1P	-5.5	LINC01864
-5.3	MTUS2-AS1	-5.4	MIR548AN	-5.8	AC105339.5	-5.5	RF00019
-5.3	PLA2G2A	-5.4	AC091180.5	-5.8	AF131215.7	-5.5	LINC01289
-5.3	INSC	-5.4	AC106872.5	-5.8	AL691447.2	-5.5	COX6CP16
-5.3	SNORD9	-5.4	AC024257.4	-5.8	PTPRT	-5.5	MYBPH
-5.3	RF00012	-5.4	AC024884.1	-5.8	AC244153.1	-5.2	PIK3R5
-5.3	RF00019	-5.4	AC130324	-5.8	NPM1P29	-5.2	NKX3-1

			.1				
-5.3	RN7SKP269	-5.4	MAP3K7CL	-5.8	AC006450.1	-5.2	CCDC92B
-5.3	RF00019	-5.4	SLCO1B3	-5.8	REV3L-IT1	-5.2	P2RX7
-5.3	MIR95	-5.4	FLJ21408	-5.8	EEF1A1P12	-5.2	FA2H
-5.3	AC009967.1	-5.4	LINC02050	-5.8	AC003991.1	-5.2	VWF
-5.3	RPL23AP53	-5.4	GRHL2	-5.5	AP001350.1	-5.2	AL031772.1
-5.3	SMPD4P1	-5.4	AC010175.1	-5.5	RNF217-AS1	-5.2	SBK2
-5.3	FCF1P1	-5.4	AC105036.2	-5.5	SLC25A21	-5.2	AC007656.1
-5.3	LINC01185	-5.4	AC091180.4	-5.5	NAMA	-5.2	GNL3LP1
-5.3	LINC01715	-5.4	RN7SL128P	-5.5	AL137784.1	-5.2	UBE2Q2P6
-5.3	PIP5K1P2	-5.4	AC124276.1	-5.5	PDIA2	-5.2	AC064850.1
-5.3	NOS2P3	-5.4	RNU2-51P	-5.5	SLC45A2	-5.2	ODF3L1
-5.3	AL138831.1	-5.4	GJB5	-5.5	TMC1	-5.2	CCDC63

-5.3	TMPRSS11CP	-5.4	MED15P5	-5.5	AL031710.2	-5.2	AP005212.1
-5.3	KRT8P15	-5.4	SLC23A3	-5.5	ISM1	-5.2	AC109992.2
-5.3	AC016745.1	-5.4	AC026191.1	-5.5	AC007326.1	-5.2	CAB39P1
-5.3	NUCB1-AS1	-5.4	RNU2-34P	-5.5	AL451007.1	-5.2	AC244090.2
-5.3	AC093151.3	-5.4	RNU6-444P	-5.5	AL590369.1	-5.2	AC022405.1
-5.3	AL445183.2	-5.4	PKP3	-5.5	AL137058.2	-5.2	AP001180.4
-5.3	Z98884.1	-5.4	FARSA-AS1	-5.5	AC020658.5	-5.2	AL031665.2
-5.3	AC003986.3	-5.4	LINC01864	-5.5	DEPDC1-AS1	-5.2	AC106864.1
-5.3	HNRNPA1P47	-5.4	RF00019	-5.5	NUDT16P1	-5.2	AC025262.1
-5.3	AC073332.1	-5.4	LINC01289	-5.5	AL135905.1	-5.2	RPL7AP2
-5.3	SNORD126	-5.4	MYBPH	-5.5	AC027288.3	-5.2	TNFRSF14-AS1
-5.3	RPL22P2	-5.1	PIK3R5	-5.5	NOC2LP1	-5.2	AC114402.1

-5.3	AL645608.4	-5.1	GAS6-DT	-5.5	CATSPER3	-5.2	AC027312.1
-5.3	RPL31P58	-5.1	ERICH2	-5.5	TFEC	-5.2	KHDC3L
-5.3	AC006387.1	-5.1	FP565260.3	-5.5	AL590282.1	-5.2	RF00019
-5.3	AC106772.1	-5.1	AC096637.2	-5.5	LINC00930	-5.2	INSC
-5.3	KNOP1P5	-5.1	AC007848.1	-5.5	AC009093.10	-5.2	MIR122HG
-5.3	AC010261.1	-5.1	AC254562.1	-5.5	UPB1	-5.2	ASB5
-5.3	AC108727.2	-5.1	SPATA9	-5.5	AL590666.4	-5.2	RNU6-335P
-5.3	RNU6-182P	-5.1	MGAT4EP	-5.5	AC104653.1	-5.2	AC008427.1
-5.3	AC010306.1	-5.1	AL109613.1	-5.5	CAGE1	-5.2	AC006387.1
-5.3	AP002981.1	-5.1	AC073136.2	-5.5	BMS1P11	-5.2	MIR8078
-5.3	AC004923.1	-5.1	AC133552.3	-5.5	AC092131.1	-5.2	AC005597.1
-5.3	AC008115.1	-5.1	AP000845.1	-5.5	NUDT19P3	-5.2	MYBPC2
-5.3	HSPA8P14	-5.1	FAM96AP	-5.5	GTF2IRD	-5.2	Z98884.1

			2		2P1		
-5.3	NT5CP1	-5.1	VCX3A	-5.5	OR7E126P	-5.2	LINC01708
-5.3	AC066612.1	-5.1	AC005670.1	-5.5	IQSEC3P1	-5.2	AC134772.1
-5.3	AC140725.1	-5.1	ADCY10	-5.5	AC024257.4	-5.2	LINC01290
-5.3	AC090907.2	-5.1	AL513548.1	-5.5	PGAM1P5	-5.2	AC104564.1
-5.3	AL032819.1	-5.1	AC027373.1	-5.5	UBE2Q2P11	-5.2	RN7SKP269
-5.3	LINC01290	-5.1	RPS20P4	-5.5	AC130324.1	-5.2	AC069439.2
-5.3	Z84723.1	-5.1	MRPL23-AS1	-5.5	AC103810.1	-5.2	CFAP299
-5.3	AL162412.1	-5.1	AC092135.3	-5.5	AC017104.3	-5.2	AL138831.1
-5.3	AC099518.4	-5.1	RNU6-1223P	-5.5	AC103681.2	-5.2	AL158198.1
-5.3	AC116914.1	-5.1	TBC1D22A-AS1	-5.5	LINC02342	-5.2	TRDN
-5.3	AC115099.1	-5.1	AC004687.2	-5.5	PMS2P7	-5.2	AP002981.1
-5.3	MIR4484	-5.1	CCDC63	-5.5	TLK2P2	-5.2	AC023794

							.4
-5.3	AC090774.1	-5.1	AC073052 .1	-5.5	FLJ21408	-5.2	AC099518 .4
-5.3	AC005546.1	-5.1	AC006378 .2	-5.5	AC003005. 2	-5.2	FCGR1A
-5.3	AC008747.1	-5.1	AC022405 .1	-5.5	AGXT	-5.2	LINC0118 5
-5.3	AL353622.2	-5.1	AC087289 .1	-5.5	AC008567. 2	-5.2	AC108727 .2
-5.3	AC026786.2	-5.1	AL354983. 1	-5.5	AC005593. 1	-5.2	AC069528 .2
-5.3	AL138831.2	-5.1	AC013400 .1	-5.5	RN7SL128 P	-5.2	LIX1-AS1
-5.3	AL356258.1	-5.1	AC005696 .3	-5.5	RNU2-51P	-5.2	GLYATL1 B
-5.3	AC004832.4	-5.1	MEI1	-5.5	AL031728. 1	-5.2	AC051619 .8
-5.3	AC005696.3	-5.1	TNFRSF1 4-AS1	-5.5	GJB5	-5.2	AC106820 .1
-5.3	AL157813.1	-5.1	AC009967 .1	-5.5	MED15P5	-5.2	SMPD4P1
-5.3	GXYLT1P4	-5.1	HCN1	-5.5	AC026191. 1	-5.2	AL645608 .4
-5.3	AC010378.1	-5.1	CDH17	-5.5	RNU2-34P	-5.2	AC016745

							.1
-5.3	AL391261.4	-5.1	AC091564.5	-5.5	RNU6-444P	-5.2	RNU6-182P
-5.3	HIST1H4A	-5.1	HSPA8P14	-5.5	RPL7L1P3	-5.2	KCNIP4-IT1
-5.3	AC069528.2	-5.1	AL135818.1	-5.5	PKP3	-5.2	AC010261.1
-5.3	AP005205.3	-5.1	AC116025.1	-5.5	COX6CP16	-5.2	KHDC1L
-5.3	KCNIP4-IT1	-5.1	AL353658.2	-5.5	MYBPH	-5.2	AC003986.3
-5.3	AC091057.7	-5.1	AC008750.3	-5.2	AP003354.1	-5.2	TMEM262
-5.3	AL354855.1	-5.1	CTXN3	-5.2	AC012603.1	-5.2	RNA5SP298
-5.3	AC127035.1	-5.1	AF186192.3	-5.2	AC021683.5	-5.2	AL355073.2
		-5.1	AC055717.1	-5.2	AL390066.1	-5.2	NOS2P3
		-5.1	DLEU7-AS1	-5.2	AL157895.1	-5.2	NUCB1-AS1
		-5.1	AC005597.1	-5.2	FAM186A	-5.2	PLA2G2A
		-5.1	MYBPC2	-5.2	AL138724.	-5.2	AC093151

					1		.3
		-5.1	ACR	-5.2	MIR4482	-5.2	HNRNPA1P47
		-5.1	AL353622.2	-5.2	KRT8P14	-5.2	MIR95
		-5.1	RPL7P8	-5.2	AC121761.1	-5.2	HIST1H4A
		-5.1	MIR3681HG	-5.2	AC011491.1	-5.2	PIP5K1P2
		-5.1	AL157791.2	-5.2	ZC3H12D	-5.2	AL354855.1
		-5.1	RF00275	-5.2	AL158834.2	-5.2	AC127035.1
		-5.1	Z98752.2	-5.2	SNX6P1	-5.2	MIR4484
		-5.1	CFAP299	-5.2	AL022157.1	-5.2	AC008115.1
		-5.1	AL158198.1	-5.2	RNU6-195P	-5.2	MGP
		-5.1	RPL37P6	-5.2	AL513314.2	-5.2	RF00019
		-5.1	AC099518.4	-5.2	AL513548.1	-5.2	AL157813.1
		-5.1	RPL31P58	-5.2	BNIP3P11	-5.2	SNORD126

		-5.1	AC115099.1	-5.2	AC026367.1	-5.2	RF00012
		-5.1	LINC01869	-5.2	TIMM9P2		
		-5.1	LINC01185	-5.2	LINC00354		
		-5.1	CERS6-AS1	-5.2	RNU6-1223P		
		-5.1	AC108727.2	-5.2	SETP12		
		-5.1	LIX1-AS1	-5.2	AL356596.1		
		-5.1	KNOP1P5	-5.2	AL157385.2		
		-5.1	GLYATL1B	-5.2	AC012409.5		
		-5.1	AC026786.2	-5.2	AL031666.2		
		-5.1	SNORD9	-5.2	AL035416.1		
		-5.1	AL391261.4	-5.2	AC109992.2		
		-5.1	AC051619.8	-5.2	AC110373.1		

		-5.1	AC090907.2	-5.2	AC025253.1		
		-5.1	Z84723.1	-5.2	AC018695.6		
		-5.1	AL645608.4	-5.2	AC087289.1		
		-5.1	AL445183.2	-5.2	AP001180.4		
		-5.1	AC016745.1	-5.2	AC013400.1		
		-5.1	RNU6-182P	-5.2	AL096678.1		
		-5.1	KCNIP4-IT1	-5.2	AC025262.1		
		-5.1	AC010261.1	-5.2	RPL7AP2		
		-5.1	KHDC1L	-5.2	AC066612.1		
		-5.1	AC073332.1	-5.2	RF00019		
		-5.1	AC003986.3	-5.2	AC037487.1		
		-5.1	TMEM262	-5.2	MIR6870		
		-5.1	RNA5SP2	-5.2	MEI1		

			98				
		-5.1	AL355073.2	-5.2	RPL21P32		
		-5.1	NOS2P3	-5.2	AC027312.1		
		-5.1	AC004832.4	-5.2	SLC26A8		
		-5.1	PLA2G2A	-5.2	C7orf33		
		-5.1	AC093151.3	-5.2	RN7SL262P		
		-5.1	HNRNPA1P47	-5.2	AC068888.2		
		-5.1	MIR95	-5.2	NT5CP1		
		-5.1	HIST1H4A	-5.2	AC016251.2		
		-5.1	PIP5K1P2	-5.2	CDC37P2		
		-5.1	AL354855.1	-5.2	AC068014.1		
		-5.1	AC127035.1	-5.2	AL353658.2		
		-5.1	MIR4484	-5.2	AC008747.1		
		-5.1	AC008115.1	-5.2	UOX		

		-5.1	MGP	-5.2	RN7SL762P		
		-5.1	RF00019	-5.2	NPM1P37		
		-5.1	AL157813.1	-5.2	NXF4		
		-5.1	SNORD126	-5.2	AF186192.3		
		-5.1	RF00012	-5.2	TREH		
				-5.2	CERS3		
				-5.2	MIR8078		
				-5.2	AC008481.1		
				-5.2	MTND5P28		
				-5.2	AL356258.1		
				-5.2	AC107308.1		
				-5.2	AL157791.2		
				-5.2	AC091057.7		
				-5.2	RF00275		
				-5.2	TRDN		

				-5.2	RPL37P6		
				-5.2	RPL31P58		
				-5.2	AC115099.<br>1		
				-5.2	LINC0186<br>9		
				-5.2	FCGR1A		
				-5.2	CERS6-AS<br>1		
				-5.2	KNOP1P5		
				-5.2	AC026786.<br>2		
				-5.2	AL391261.<br>4		
				-5.2	AC106820.<br>1		
				-5.2	AL445183.<br>2		
				-5.2	KHDC1L		
				-5.2	AC073332.<br>1		
				-5.2	TMEM262		
				-5.2	RNA5SP2<br>98		

				-5.2	AL355073.2		
				-5.2	NUCB1-AS1		
				-5.2	AC004832.4		
				-5.2	PLA2G2A		
				-5.2	AC093151.3		
				-5.2	HNRNPA1P47		
				-5.2	MIR95		
				-5.2	HIST1H4A		
				-5.2	PIP5K1P2		
				-5.2	AL354855.1		
				-5.2	AC127035.1		
				-5.2	MIR4484		
				-5.2	AC008115.1		
				-5.2	MGP		
				-5.2	RF00019		

				-5.2	AL157813.1		
				-5.2	SNORD126		
				-5.2	RF00012		
				-5.0	AL513523.2		

Supplementary Table 2 Antibodies used in this study

Supplementary Table 2

The antibodies used in this study

Antigen Name	Host	Clonality	Manufacturer	I.D.	Dilution
Nestin	Mouse	monoclonal	Santacruz	23927	1:100
P2X7 (Immunohistochemistry)	Rabbit	polyclonal	Proteintech	28207-1-AP	1:100
P2X7 (western blot)	Mouse	monoclonal	Santacruz	514962	1:1000

Product name	Host	Clonality	Manufacturer	I.D.	Dilution
mouse IgG(H+L) AlexaFluo488	Donkey	polyclonal	Thermo Scientific	A21202	1:300
rabbit IgG(H+L) AlexaFluo488	Donkey	polyclonal	Thermo Scientific	A21206	1:300
goat IgG(H+L) HRP	Horse	polyclonal	Cell signaling	7076	1:1000

Supplementary Table 3 Primers used in this study

Supplementary Table 3

Primers for qPCR

gene name		Sequence (from 5' to 3')
nestin	Fw	GAAGGGCAATCACAACAGGTG
	Rv	GGGGCCACATCATCTTCCA
p2x7	Fw	GTGCCGAAAACTTCACTGTGC
	Rv	CTGGCAGGATGTTTCTCGTGG
ribosomal protein 18s	Fw	GCGGCGGAAAATAGCCTTTG
	Rv	GATCACACGTTCCACCTCATC
p2Rx3	Fw	GCGTTTCTGAGAAAAGCAGCGTG
	Rv	CGGATGCCAAAAGCCTTCAGGA
p2Rx4	Fw	GTGGCGGATTATGTGATACCAGC
	Rv	CACACAGTGGTCGCATCTGGAA
p2Rx5	Fw	CGCTTTGACGTGATGGTGAACG
	Rv	TCCTGGGAACTGTCTTCTAGGC
p2Rx6	Fw	AAGCACTGCCGCTATGAACCAC
	Rv	CCAGTGAACTCTGATGCCTACAG
p2Ry1	Fw	GCCATCTGGATGTTCGTCTTCC
	Rv	TGGCAGAGTCAGCACGTACAAG
p2Ry2	Fw	CGAGGACTTCAAGTACGTGCTG
	Rv	GTGGACGCATTCCAGGTCTTGA
p2Ry11	Fw	AGCAGAAACGGCTTGCCAAGTC
	Rv	GAACCCACTGAGTTTGTCGTCG

Supplementary Figures

Supplementary Figure 1 Whole blot images of Figure 2 a

Raw luminescent images were taken, and automatically merged with the visual light images by the FUSION Solo S software. As the internal control, we detected beta-Actin protein using the same membrane reproved by stripping solution.

Supplementary Figure 2 Gene expression levels of the p2x and p2y families in iNSCs

(a) The mRNA expression levels of p2x3, 4, 5, and 6 in iNSCs in comparison with the undifferentiated iPS cells. (b) The mRNA expression levels of p2y1, 2, and 11 in iNSCs in comparison with the undifferentiated iPS cells. Please note that the expressions of the above subtypes are also observed in the global RNA sequencing in Figure 3.

Supplementary Figure 3 Inhibitory effect of the P2X7-specific antagonist JNJ-47965567 and AZD9056 in ATP-induced calcium sparks

Representative calcium sparks of the three cells were shown.

Supplementary Figure 4 MLM-2 sorted noneducated haloperidol and DHA but not GABA into the positive group

(**a**) MLs were performed using composite images obtained from newly cultured iNSCs from master stocks for education. (**b**) Prediction of haloperidol, DHA, and GABA as new substances theoretically belonging to the positive group.

Supplementary Figure 5 General statistical analyses of waveforms

(**a**) Frequency. (**b**) Dynamic range. Unpaired student-*t* tests were performed by the software Microsoft Excel. N.S. means statistically not significant ($p>0.05$).

Supplementary Figure 6 MLM-3 using selected waveforms with a smaller dynamic range

(**a**) MLs were performed using single-waveform image with similar dynamic ranges for education. (**b**) MLs were performed using composite images (nine waveforms with similar dynamic ranges in one image) for education.

Supplementary Figure 7 Similar prediction abilities of MLMs developed using the same number of waveforms in single and composite

(**a**) MLM developed using negative 1152 and positive 1017 single waveforms. (**b**) MLM developed using negative128 and positive 113 composite-images containing 1152 and 1017 waveforms, respectively.

Supplementary Figure 8 The properties of initial ATP-induced calcium sparks in iNSCs

We observed intracellular calcium concentrations in the six iNSCs before and after ATP addition. In each case, fluorescent intensity was shown in f/f0, which was calculated by dividing by the initial fluorescent intensity. Please note that after the addition of ATP, fluorescent signals from all cells showed rapid increases and, afterward, gradual decreases.

Supplementary Video 1 Calcium sparks of iNSCs induced by ATP without BBG

To visualize time laps images, the playback speed was modified artificially to increase

by 32-times.

Supplementary Video 2 Calcium sparks of iNSCs induced by ATP with 20μM BBG

To visualize time laps images, the playback speed was modified artificially to increase by 32-times.

Supplementary Figure 1

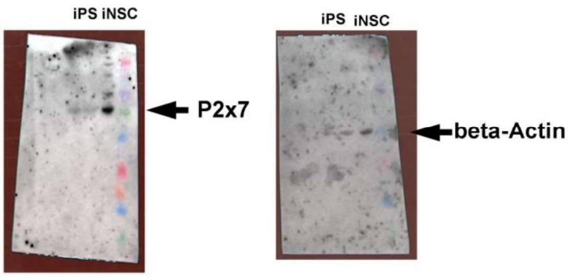

Supplementary Figure 2

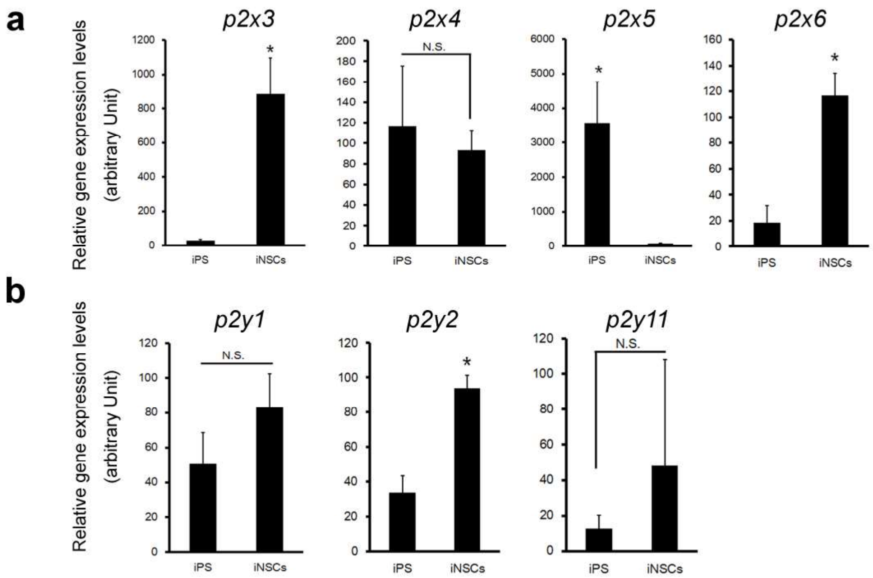

Supplementary Figure 3

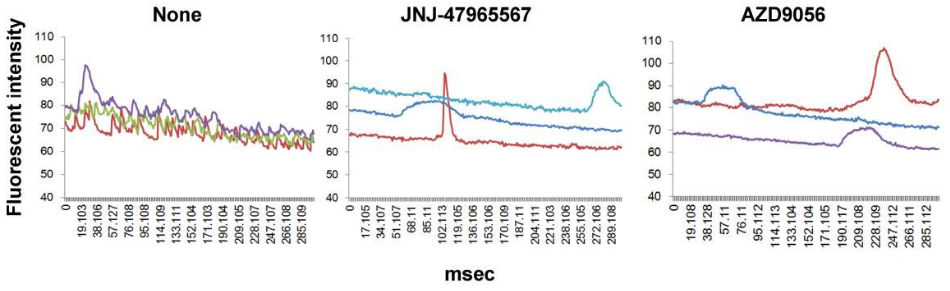

Supplementary Figure 4

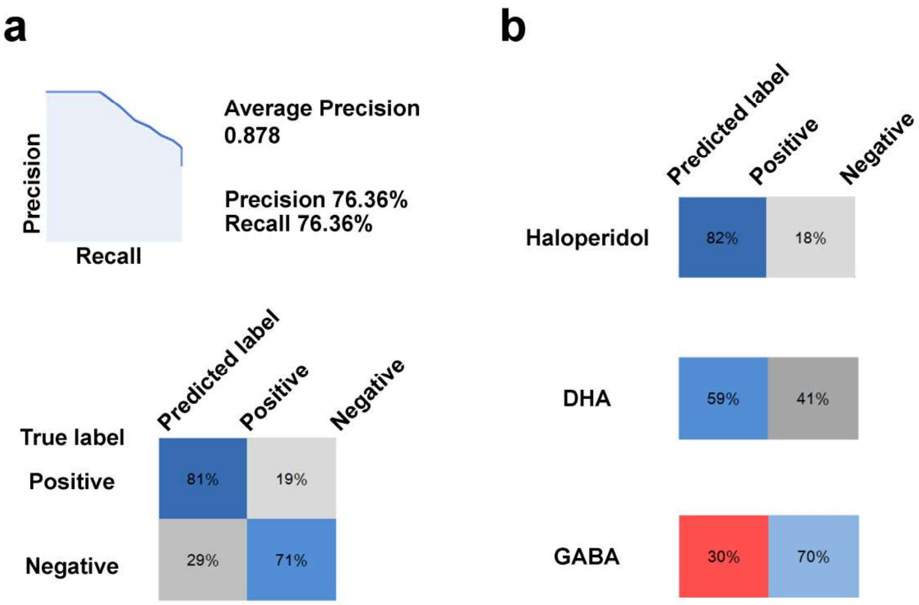

Supplementary Figure 5

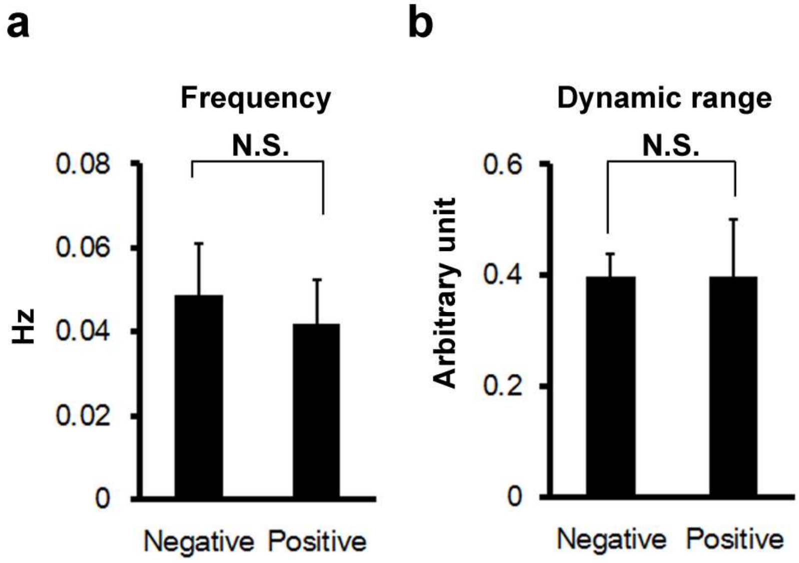

Supplementary Figure 6

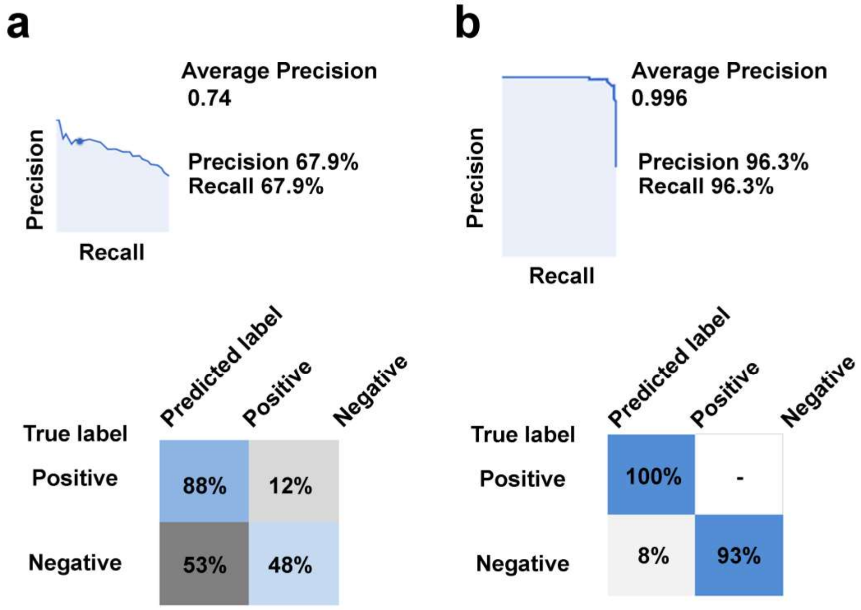

Supplementary Figure 7

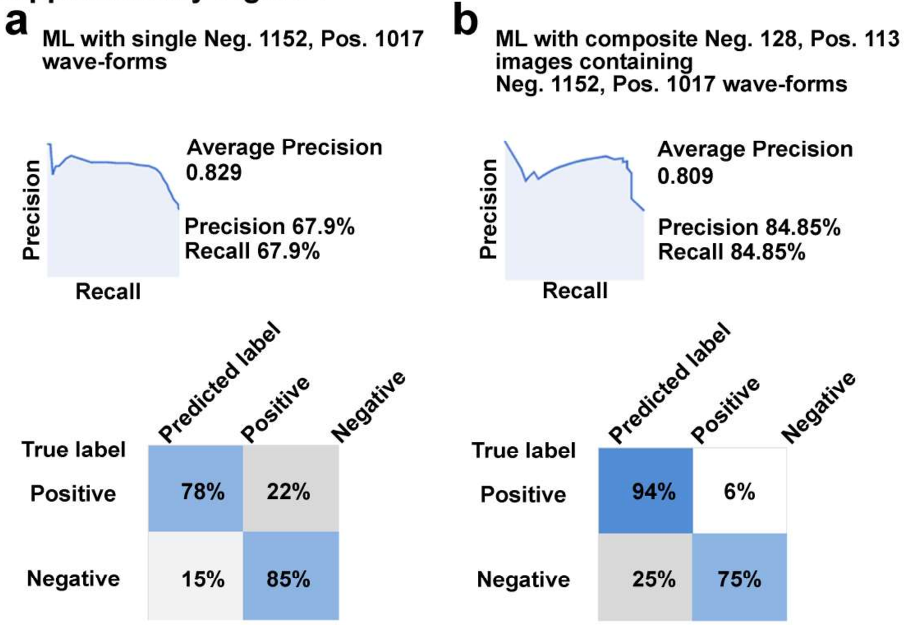

Supplementary Figure 8

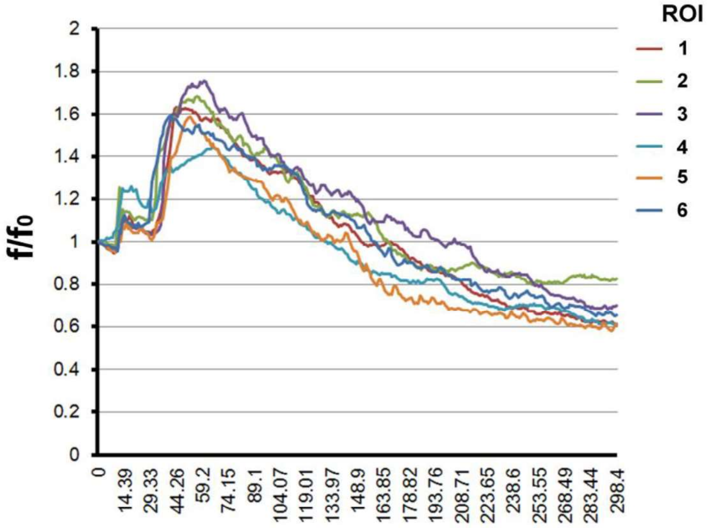

Supplement: Supplementary file 1 — Supplementary Information 1. [file 41598_2023_39846_MOESM1_ESM.pdf]
